# Epidemiology and control of SARS-CoV-2 epidemics in partially vaccinated populations: a modeling study applied to France

**DOI:** 10.1186/s12916-022-02235-1

**Published:** 2022-01-26

**Authors:** Paolo Bosetti, Cécile Tran Kiem, Alessio Andronico, Vittoria Colizza, Yazdan Yazdanpanah, Arnaud Fontanet, Daniel Benamouzig, Simon Cauchemez

**Affiliations:** 1 Mathematical Modelling of Infectious Diseases Unit, Institut Pasteur, Université de Paris, UMR2000, CNRS, Paris, France; 2 Collège Doctoral, Sorbonne Université, Paris, France; 3 INSERM, Sorbonne Université, Institut Pierre Louis d’Epidémiologie et de Santé Publique, Paris, France; 4 Université of Paris, INSERM UMR 1137 IAME, Paris, France; 5 Department of Infectious Diseases, Assistance Publique-Hôpitaux de Paris, Bichat–Claude-Bernard University Hospital, Paris, France; 6 Emerging Diseases Epidemiology Unit, Université de Paris, Institut Pasteur, Paris, France; 7 PACRI Unit, Conservatoire National des Arts et Métiers, Paris, France; 8 Sciences Po - Centre de sociologie des organisations and Chaire santé – CNRS, Paris, France

**Keywords:** SARS-CoV-2, Vaccination, Non-pharmaceutical interventions

## Abstract

**Background:**

Vaccination is expected to change the epidemiology and management of SARS-CoV-2 epidemics.

**Methods:**

We used an age-stratified compartmental model calibrated to French data to anticipate these changes and determine implications for the control of an autumn epidemic. We assumed vaccines reduce the risk of hospitalization, infection, and transmission if infected by 95%, 60%, and 50%, respectively.

**Results:**

In our baseline scenario characterized by basic reproduction number R_0_=5 and a vaccine coverage of 70–80–90% among 12–17, 18–59, and ≥ 60 years old, important stress on healthcare is expected in the absence of measures. Unvaccinated adults ≥60 years old represent 3% of the population but 43% of hospitalizations. Given limited vaccine coverage, children aged 0–17 years old represent a third of infections and are responsible for almost half of transmissions. Unvaccinated individuals have a disproportionate contribution to transmission so that measures targeting them may help maximize epidemic control while minimizing costs for society compared to non-targeted approaches. Of all the interventions considered including repeated testing and non-pharmaceutical measures, vaccination of the unvaccinated is the most effective.

**Conclusions:**

With the Delta variant, vaccinated individuals are well protected against hospitalization but remain at risk of infection and should therefore apply protective behaviors (e.g., mask-wearing). Targeting non-vaccinated individuals may maximize epidemic control while minimizing costs for society. Vaccinating children protects them from the deleterious effects of non-pharmaceutical measures. Control strategies should account for the changing SARS-CoV-2 epidemiology.

**Supplementary Information:**

The online version contains supplementary material available at 10.1186/s12916-022-02235-1.

## Background

The SARS-CoV-2 pandemic that started in December 2019 has caused more than 5 million deaths around the world and led healthcare systems at the brink of collapse in many countries. In addition, the drastic control measures that were implemented to limit its impact have had dramatic socio-economic consequences.

Vaccines have proved effective at reducing the severity of SARS-CoV-2 infection [1], the risk of infection [2], and transmission [3]. A number of modeling studies evaluated how vaccination will help mitigate a SARS-CoV-2 epidemic rebound this autumn, highlighting that it might be difficult to fully relax control measures given the high transmissibility and severity of SARS-CoV-2 [4–7]. These studies assessed the impact on key health metrics (e.g., number of hospitalizations and death) and identified the level of social distancing that would remain necessary as a function of vaccine coverage. A question that has received less attention is that, in this new era where a large part of the population is vaccinated, the epidemiology of SARS-CoV-2 (Who is infected? Who transmits? Who is hospitalized?) will be different from what it was prior to the distribution of vaccines [8]. It is important to anticipate these changes to determine how control measures might evolve to ensure they maintain the epidemic under control while minimizing costs for society. For example, the expectation that unvaccinated individuals will have a higher contribution to infections, transmissions, and hospitalizations has led some countries to introduce control strategies specifically targeting this population. This is the case of France: confronted to a rapid rise in Delta cases and a plateau in vaccinations in June–July 2021, French authorities announced in July that a sanitary pass, i.e., a proof of completed vaccination, recent infection or recent negative test, would be required to access places such as bars, restaurants, and cinemas. The announcement led to an important surge in vaccination appointments and in vaccine coverage. A number of European countries introduced similar measures.

Here, we developed a mathematical model to characterize the epidemiology of SARS-CoV-2 in a partially vaccinated population and evaluate in this new context the contribution to transmission and healthcare burden of individuals of different ages and vaccination status. This information is used to ascertain different control strategies, including repeated testing and non-pharmaceutical measures, targeting the whole population or subgroups such as unvaccinated individuals to optimally mitigate an autumn epidemic rebound. This is also an opportunity to revisit impact assessment accounting for the increased transmissibility and severity of the Delta variant as well as the reduction in vaccine protection against infection associated with this variant. We consider Metropolitan France as a case study.

## Methods

### Deterministic model

We developed a deterministic age-stratified compartmental model describing the spread of SARS-CoV-2 in metropolitan France. The model, which accounts for French age-specific contact patterns [9], has been described in detail elsewhere [10]. It accounts for a gradient of severity with age [11], assuming that Delta VOC is 50% more severe than Alpha VOC [12] while Alpha VOC is 40% more severe than previously circulating strains [13]. It has been extended to account for the roll-out of vaccines [4] as well as the deployment of self-administered rapid antigenic tests [14]. A full description of the model and equations is reported in the Additional file 1 [15].

### Scenarios

#### Vaccine coverages and characteristics

Considering the Delta variant, we assume that vaccines are 95% effective at reducing the risk of hospitalization [1], 60% at reducing the risk of infection [16] (impact on susceptibility), and 50% at reducing the infectivity of vaccinated individuals [3]. In a sensitivity analysis, we show results if vaccines are 80% effective at reducing the risk of infection [2] (which was the scenario considered prior to the rise of Delta) and 90% effective against hospitalization. We build several scenarios regarding vaccine coverage achieved in the different age groups by September 1st, 2021: 90% or 95% among those older than 60 years old; 60%, 80%, or 90% among those aged 18–59 years old and 0%, 30%, or 70% among the 12–17 years old (called teenagers in the following). To give some context, 89% of those older than 60 years old, 84% of the 18-59 years old and 61% of the 12-17 years old have received a first dose of vaccines against SARS-CoV-2 by August 25th, 2021. In our baseline scenario that we label 70–80–90%, we assume vaccination coverage will reach 70%, 80%, and 90% among 12–17 years old, 18–59 years old, and over 60 on September 1st, 2021. In this analysis, we consider that the vaccine coverage corresponds to the proportion of the population having acquired vaccine protection after two doses if required.

#### Epidemic dynamics with and without control measures

We assume that, by September 1st, 2021, 25% (range: 20–30%) of the French population has been infected by SARS-CoV-2 (see Additional file 1), benefiting from natural protection against reinfection. We then explore scenarios where different types of control measures are implemented.

First, we explore scenarios where control measures are completely relaxed in the Autumn. These scenarios are characterized by the basic reproduction number *R*_0_, i.e., the average number of persons infected by a case in a population with no immunity and no control measures. In March 2020, *R*_0_ was estimated at around 3 in France prior to the implementation of a nation-wide lockdown [10]. The emergence of more transmissible variants of concerns (VOC) (such as the Alpha and Delta VOCs) [17–20] is expected to increase *R*_0_. We therefore explore scenarios in which *R*_0_ ranges between 3.0 and 6.0 when measures are completely relaxed, considering *R*_0_=5 as our baseline scenario. We assume that from September 1st, 2021, the structure of contacts in the population comes back to the one measured during the pre-pandemic period [9].

We then consider scenarios where different types of control measures are implemented, targeting different groups:

- Random testing: we assume that a proportion of the population is targeted for random testing with antigenic tests. These individuals test at regular intervals (every 7 days in the baseline scenario; twice a week and every 2 weeks in sensitivity analyses). We assume that individuals testing positive isolate in a way that reduces onward transmission by 75%. We consider a scenario where 50% of unvaccinated individuals aged ≥12 years old get tested and a scenario where the same number of individuals randomly drawn among individuals aged ≥12 years old (vaccinated or unvaccinated) are tested. We consider scenarios where the antigenic test is performed by the individual (self-swabbing and reading of the result; sensitivity: 75%) or by a professional (sensitivity 90%). In a sensitivity analysis, we also explore a scenario where 25% of unvaccinated individuals ≥12 years old get tested.
- Non-pharmaceutical interventions: Non-pharmaceutical interventions such as social distancing, protective measures and mask-wearing may be used to reduce transmission rates. We consider scenarios where such measures target the whole population, leading to reductions of transmission rates of 10%, 20%, 30%, or 40% from any infected individual, whether they have been vaccinated or not. We also consider scenarios where such measures only target unvaccinated individuals, leading to reductions of transmission rates of 10%, 20%, 30%, or 40% from unvaccinated individuals, while transmission rates from vaccinated individuals remain unchanged.
- Increased vaccine coverage among unvaccinated individuals: We compare the performance of these interventions to that obtained if 50% of the unvaccinated individuals aged ≥12 years old were to get vaccinated.

Children are defined as individuals aged 0–17 years old We assume that children aged 0–9 years old are 50% less susceptible to infection than adults while those aged 10–17 years old are 25% less susceptible to infection than adults [10, 21]. In a sensitivity analysis, we also assume that children aged 0–9 years old are 50% less infectious than adults [22].

We assume an antigenic test costs 5 euros if performed by the individual, 11 euros if performed by a professional, and a 2-dose vaccine costs 32 euros. Models are run until March 20th, 2022.

## Results

### Baseline scenario and no control measures

We first present results under the assumption that control measures are completely relaxed in autumn 2021, for our baseline scenario (basic reproduction number *R*_0_=5 and a vaccine coverage of 70–80–90% among teenagers, adults aged 18–59 years old and over 60, respectively). In this case and assuming that 25% of the population acquired immunity through infection by September 1st, our model anticipates a wave characterized by a peak of 5200 hospital admissions per day which is larger than the peaks observed in France during the two pandemic waves of 2020. The peak would be at 7300 and 3400 admissions per day if the proportion infected by September 1st was 20% and 30%, respectively.

**Fig. 1:**
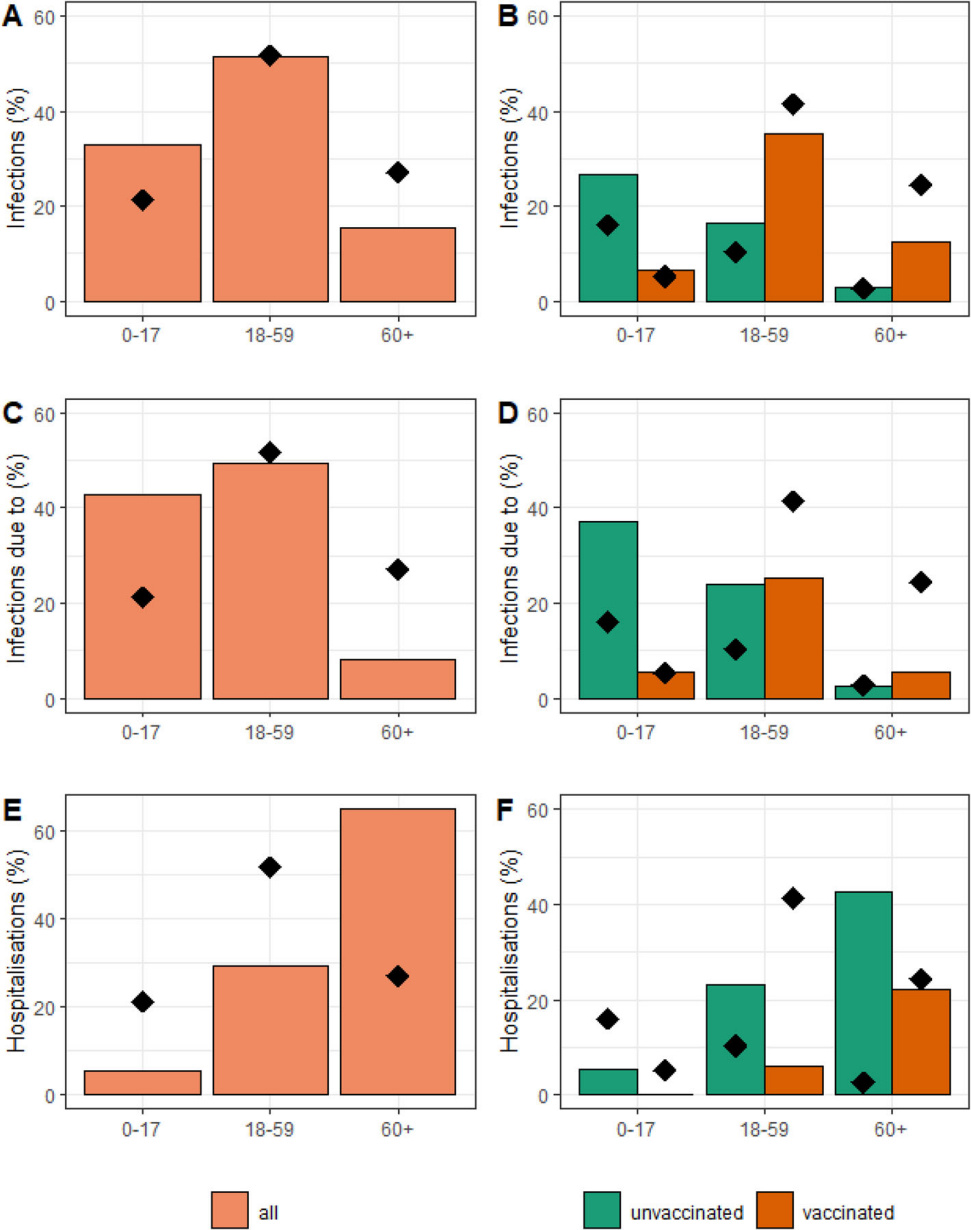
Contribution of groups defined by their age and vaccination status to infections, disease spread and hospital burden, in our baseline scenario with *R*_0_=5 and a vaccine coverage of 70–80–90% among 12–17 years old, 18–59 years old, and over 60 years old. Age distribution of new infections (**A)** in the entire population and (**B)** among vaccinated and unvaccinated individuals. Proportion of infections (**C)** attributable to different age groups and (**D)** attributable to different age groups among vaccinated and unvaccinated individuals. Age distribution of hospitalizations (**E)** in the entire population and (**F)** among vaccinated and unvaccinated individuals. In all panels, the diamonds indicate the age distribution of the different groups in the population

We anticipate that the roll-out of vaccines will modify the epidemiology of SARS-CoV-2. In a context where most adults are vaccinated but vaccine coverage remains limited among children (0–17 years old), we expect 33% of infections will occur in this age group, even though they only represent 22% of the population and are assumed to be less susceptible to SARS-CoV-2 infection than adults (Fig. 1A). In each age group, unvaccinated individuals are overrepresented among infected people while vaccinated individuals are under-represented (Fig. 1B). For example, the risk of infection for an unvaccinated individual is RR=1.9 times higher than that of a vaccinated individual among those aged 18–59 years old (RR=1.3 among 0–17 years old and RR=2.2 among over 60; Additional file 1: Table 1). Overall, unvaccinated individuals represent 29% of the population but 46% of infections. Their contribution to the transmission process is even higher with a risk of transmission from an unvaccinated individual that is 4.3 times higher than that from a vaccinated individual (Fig. 1C, D).

Vaccination will also impact the age distribution of those hospitalized. While 74% of hospitalizations occurred among those older than 60 years old in the pre-vaccination era, this proportion is expected to drop to 65% in our baseline scenario. In parallel, the proportion of 18–59 years old among hospitalized individuals increases from 25% in the pre-vaccination era to 30% (Fig. 1E). The small group of unvaccinated adults that are older than 60 years old has a disproportionate impact on the stress to the healthcare system. They represent 10% of their age group but 66% of hospitalizations from that age group (RR: 17.2), and 3% of the general population but 43% of all hospitalizations (RR: 26.7) (Fig. 1F). Even though we assume that the vaccine is 95% effective against the risk of hospitalization, in a context where vaccine coverage is high among older individuals, 28% of hospitalizations occur among vaccinated people (Fig. 1F).

### Baseline scenario with control measures

**Fig. 2:**
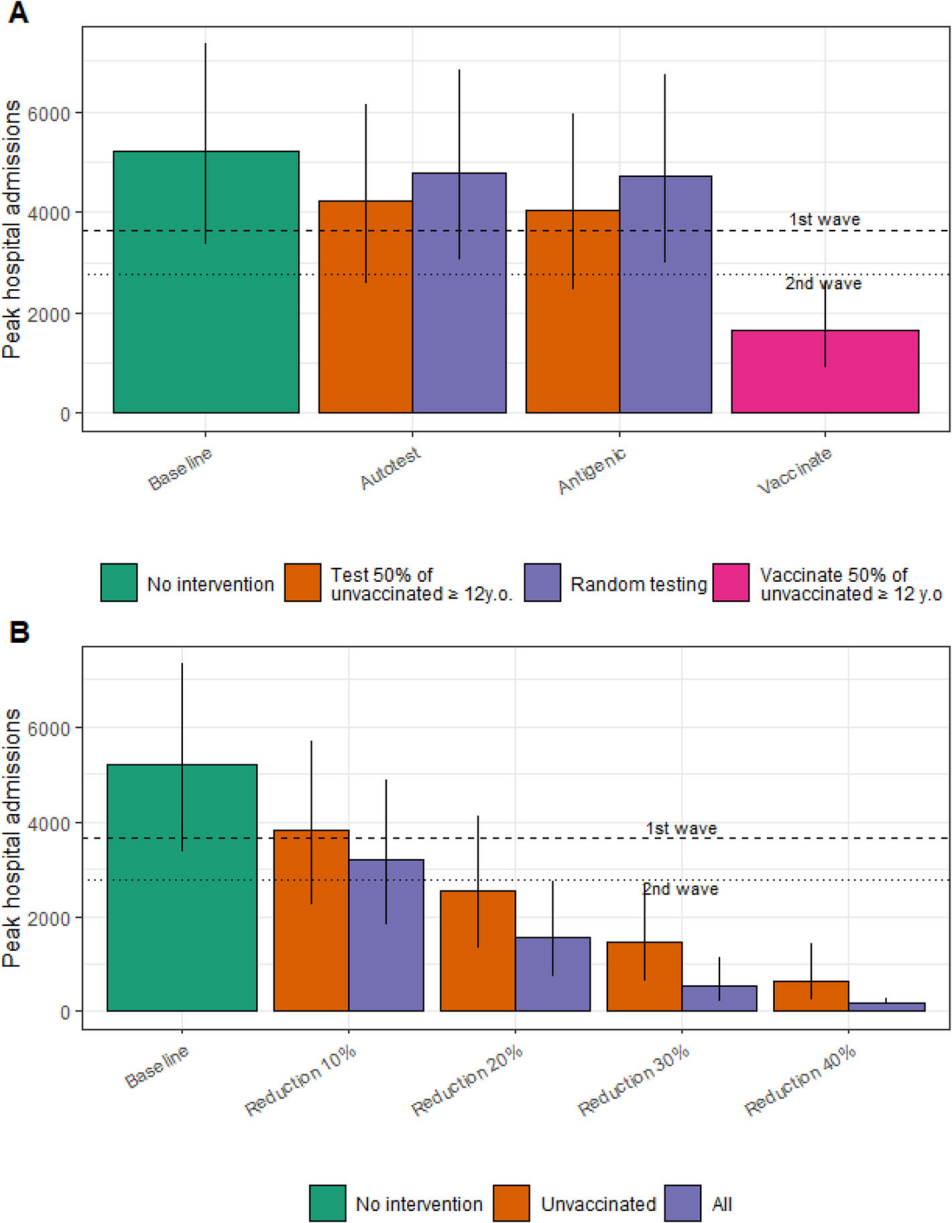
Comparison of the impact of control strategies targeting the entire population vs unvaccinated individuals only, in our baseline scenario with *R*_0_=5 and a vaccine coverage of 70–80–90% among 12–17 years old, 18–59 years old, and over 60 years old. (**A)** Peak in daily hospital admissions under different testing strategies. *Baseline*, no intervention; *Autotest unvaccinated*, 50% of the unvaccinated individuals aged ≥12 years old are tested weekly (sensitivity of 75%); *Autotest random*, the same number of individuals as in the *Autotest unvaccinated* are tested but among individuals aged ≥12 years old, irrespective of vaccine status; *Antigenic unvaccinated*, same as in *Autotest unvaccinated* but with tests performed by a professional (sensitivity of 90%); *Antigenic random*, same as in *Autotest random* but with tests performed by a professional (sensitivity of 90%); *Vaccinate*, 50% of the unvaccinated individuals aged ≥12 years old are vaccinated. **B** Peak in daily hospital admissions under non-pharmaceutical interventions of varying intensities. *Baseline*, no intervention; *Reduction of x% unvaccinated*, The transmission rate of unvaccinated individuals is reduced by *x*%; *Reduction of x% all*, The transmission rate at the population level is reduced by *x*%. We assume 25% of the population has acquired protection through natural infection (range 20–30% corresponding to the vertical bars)

We then investigate the impact of different control strategies targeting different groups for our baseline scenario with a vaccination coverage 70–80–90% and *R*_0_=5. Weekly testing of 50% of unvaccinated individuals aged ≥12 years old could reduce the peak of hospitalizations by 19% (range: 16–23% for 20–30% of the population infected prior to September 1st, 2021) if an autotest is used and 22% (19–27%) if the test is performed by a professional (Fig. 2A). In contrast, if the same number of tests were distributed randomly among individuals aged ≥12 years old irrespective of vaccination status, the reductions in hospital admissions would only be of 8% (7–10%) and 10% (8–12%), respectively. The reduction in the peak of hospitalizations would be much larger if 50% of unvaccinated individuals aged ≥12 years old agreed to get vaccinated instead of being repeatedly tested (68% vs 19%; Fig. 2A), for a cost that would be 4.5 times lower (0.16 vs 0.72 billion euros; Additional file 1: Fig. S2). Moreover, only vaccination would be able to reduce the peak of hospital admissions below the peaks observed in the 2020 spring and fall waves.

Non-pharmaceutical interventions applied to all and reducing the overall transmission rates by 10%, 20%, 30%, and 40% would reduce the peak of hospitalizations by 39%, 70%, 90%, and 97%, respectively (Fig. 2B). If these measures were only targeted towards the few unvaccinated individuals (29% of the population), we could still reach 27%, 51%, 72%, and 87% peak reductions, respectively. In both scenarios, reducing transmission rates by 20% would be sufficient to make the peak of hospitalizations drop below the levels observed during the second wave of 2020. Given the residual risk of infection in vaccinated individuals with the Delta variant, substantial gains can be made if vaccinated individuals mitigate their risk of infection for example by wearing masks.

### Sensitivity analyses

**Fig. 3:**
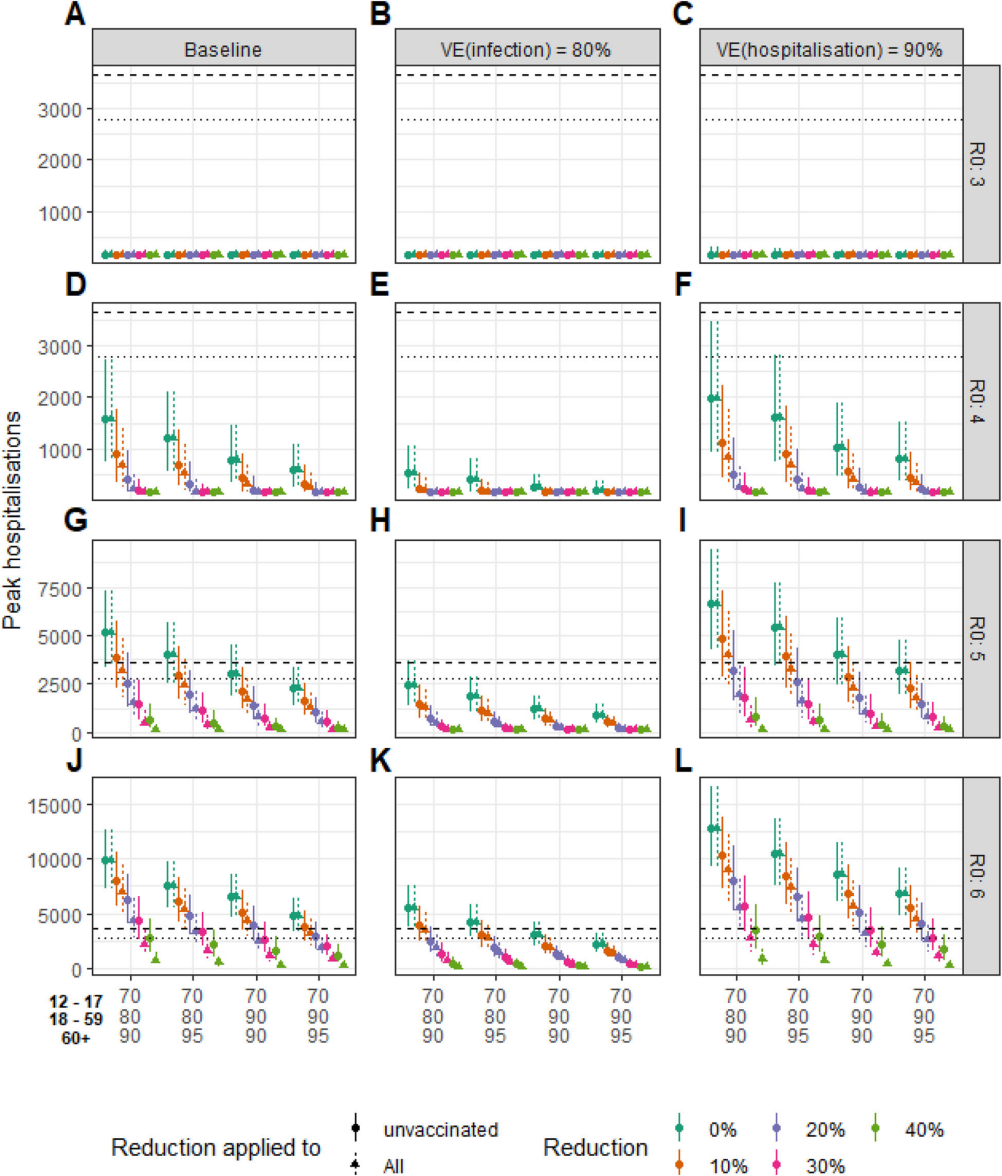
Expected size of the peak of hospitalizations when non-pharmaceutical interventions target unvaccinated individuals only or the whole population, as a function of the basic reproduction number *R*_0_, vaccine coverage in the 12–17 years old, 18–59 years old, and over 60 years old and for different efficacy of the vaccine against the risk of infection or hospitalization. Non-pharmaceutical interventions reduce the transmission rate of unvaccinated individuals (points) or the whole population (triangles) by 0%, 10%, 20%, 30%, and 40%. *R*_0_ takes the values 3.0 (top row, **A**, **B**, **C**), 4.0 (**D**, **E**, **F**), 5.0 (**G**, **H**, **I**), and 6.0 (bottom row, **J**, **K**, **L**). In the baseline scenario (left column) we assume that the vaccines are 95% effective at reducing the risk of hospitalization, 60% at reducing the risk of infection, and 50% at reducing the infectivity of vaccinated individuals. In sensitivity analyses, we consider an 80% reduction against infection (middle column) and a 90% reduction against hospitalization (right column). We assume 25% of the population has acquired protection through natural infection (range 20–30% corresponding to the vertical bars). Horizontal lines indicate the peak of daily hospital admissions observed during the first (dashed line) and the second (dotted line) epidemic wave of 2020

**Fig. 4:**
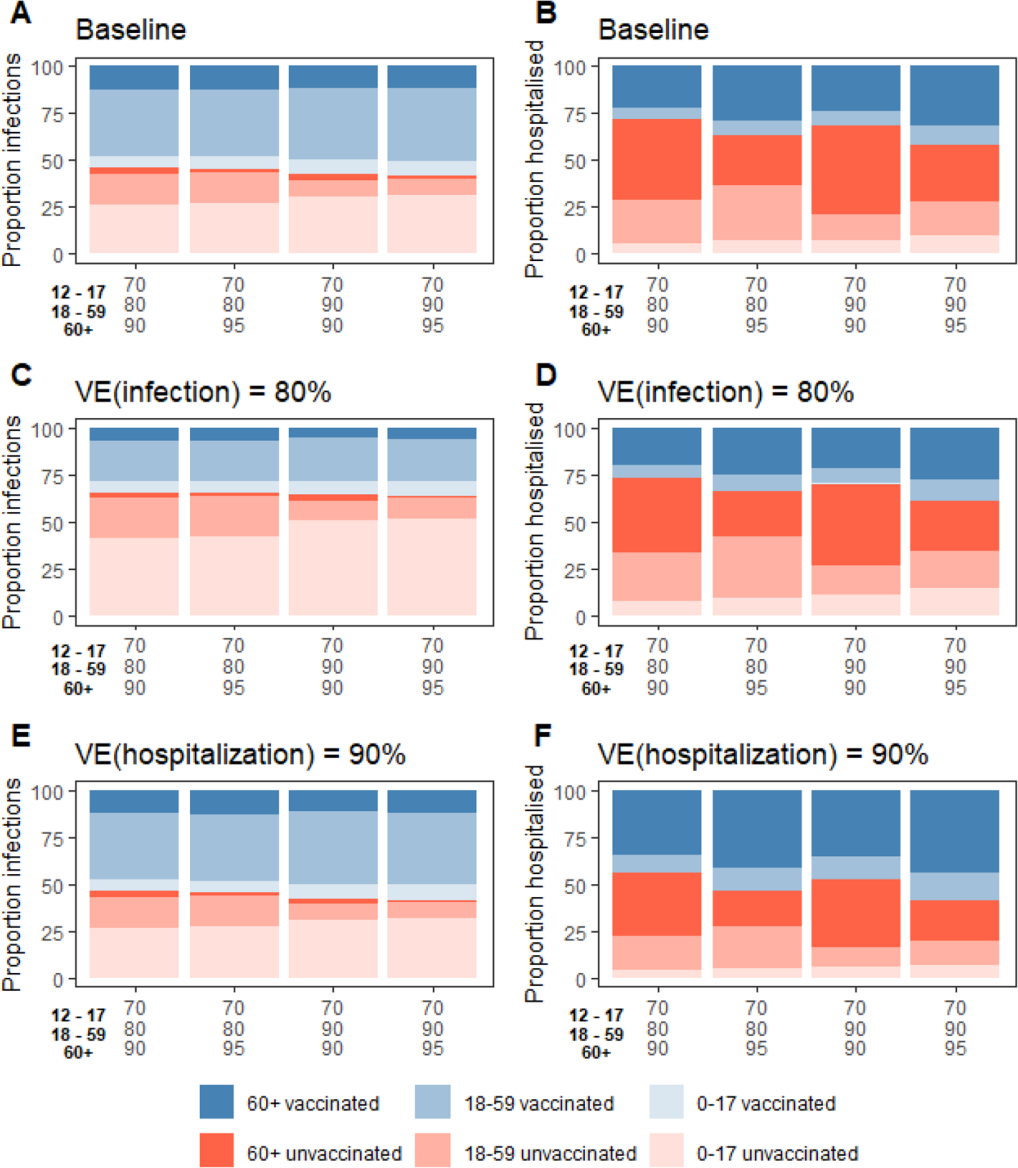
Proportion of infections (**A**, **C**, **E**) and hospitalizations (**B**, **D**, **F**) among groups defined by their age and vaccination status as a function of the vaccine coverage in the 12–17 years old, 18–59 years old, and over 60 years old. In the baseline scenario (**A**, **B**), we assume that vaccines are 95% effective at reducing the risk of hospitalization, 60% at reducing the risk of infection and 50% at reducing the infectivity of vaccinated individuals. In (**C** and **D)**, we assume a vaccine efficacy at reducing the risk of infection of 80%. In (**E** and **F)**, we assume a vaccine efficacy at reducing the risk of hospitalization of 90%. The distribution is reported for infections and hospitalizations occurring between September 1st, 2021, and March 20th, 2022 (end of the study period), for *R*_0_=5.0. We assume 25% of the population has acquired protection through natural infection

Keeping in mind that uncertainties remain about *R*_0_ and the vaccine coverage in the Autumn, we investigate how our results change if we depart from our baseline assumptions. Figure 3 shows the expected size of the autumn peak in hospital admissions, for different values of *R*_0_ and vaccine coverages, considering scenarios where control measures can target unvaccinated individuals only or the whole population leading to reductions of transmission rates in the targeted population between 0% (no control) and 40%. As expected the size of the peak increases with *R*_0_ and declines with the vaccination coverage. For *R*_0_=3, which was the value estimated for the historical lineage, we would not anticipate an epidemic rebound for the vaccine coverage expected to be achieved in France. For *R*_0_=4, the peak is expected to be below the ones of 2020 even if measures are fully relaxed. For *R*_0_=5, in the absence of control measures, the peak could remain below the one of the autumn 2020, if vaccine coverage was increased to 70% in teenagers, 90% in 18–59 years old, and 95% in those over 60 years old. For *R*_0_=6, increasing the vaccine coverage to these levels would still not allow a full relaxation of control measures and the implementation of control measures would be necessary to further mitigate the impact on healthcare. Overall, high vaccination coverage and even limited control of transmission can help mitigate an epidemic rebound. The age distribution of infected and hospitalized individuals depends on vaccine coverage in the different age groups (Fig. 4, Additional file 1: Fig. S3-S4-S10-S11). For example, when vaccine coverage in those over 60 years old increases from 90 to 95%, the contribution of this age group to hospitalizations drops from 65 to 55%. Those distributions are relatively robust to a change in *R*_0_ (Additional file 1: Fig. S3-S4).

Comparing our baseline scenario (60% reduction in the risk of infection given Delta) to that with an 80% reduction in the risk of infection that was considered prior to the rise of Delta, we find that the lower protection conferred by vaccines against Delta infection substantially degrades projections, with a peak of hospitalizations that roughly doubles when moving from 80 to 60% protection (Fig. 3). This reduction of protection against infection also increases the contribution of vaccinated individuals to infections: they represent 34% of infections with a protection of 80% compared to 54% with a protection of 60% (Fig. 4, Additional file 1: Fig. S7). As a consequence, compared to vaccinated individuals, unvaccinated individuals are 4 and 10 times more likely to transmit in scenarios with a protection against infection of 60% and 80%, respectively, for *R*_0_=5 and a vaccine coverage of 70–80–90%.

For 90% protection against hospitalization instead of 95% in our baseline scenario, we expect a 28% increase in the peak of hospitalizations (Fig. 3). This also increases the proportion of vaccinated individuals among those hospitalized from 28 to 44% (Fig. 4, Additional file 1: Fig. S8).

If we assume that vaccinated individuals that get infected have the same probability to transmit as unvaccinated individuals (compared to a 50% reduction in our baseline scenario), the contribution of vaccinated individuals to transmission increases (Additional file 1: Fig. S9). In this scenario, the probability of transmission from an unvaccinated person is 1.7 times higher than that from a vaccinated person (compared to 4.3 with the baseline assumption).

Comparing our baseline scenario (70% of teenagers vaccinated) to one where teenagers are not vaccinated (Additional file 1: Fig. S12), our results suggest that the vaccination of teenagers may substantially reduce the stress on the healthcare system. For example, if 80% of 18–59 years old and 90% of over 60 are vaccinated, the vaccination of 70% of teenagers could reduce the peak of hospitalizations by 66% and 40% for *R*_0_=4 and 5, respectively, compared to a scenario where they are not vaccinated.

If children aged 0–9 years old are 50% less infectious than adults in addition to being 50% less susceptible, the proportion of children among infections decreases from 33 to 31% while the proportion among those that cause infection drops from 43 to 36% (Additional file 1: Fig. S5). If children aged 0 to 17 years old were as susceptible as adults, the proportion of infections in this age group might reach 44% (compared to 33% in the baseline scenario) (Additional file 1: Fig. S6). Furthermore, in our baseline scenario (with vaccine coverage of 70–80–90% among 12–17, 18–59, and 60 years old and *R*_0_ = 5), the vaccination of 30% and 50% of children aged 5 to 11 years old can reduce the peak in hospital admissions by 20% and 33%, respectively (Additional file 1: Fig. S13).

## Discussion

Countries with partially vaccinated populations enter a new era in the control of the SARS-CoV-2 epidemic. However, given the high transmissibility and severity of the Delta variant and the reduced efficacy of vaccines against infection by this variant, SARS-CoV-2 may continue to generate substantial stress on healthcare in the absence of mitigation measures, even with high vaccine coverage. Nonetheless, the partial vaccination of the population modifies the epidemiology of SARS-CoV-2. Here, we used a mathematical model applied to Metropolitan France to anticipate these changes and determine how control measures might evolve in the autumn of 2021 to maximize their impact while minimizing costs.

This autumn, the stress on the healthcare system in the absence of any control measures will depend on the vaccine coverage and the transmission potential *R*_0_ of the dominant variant. *R*_0_ was around 3 for the historical lineages [10]. The Alpha variant was found to be about 50% more transmissible than historical lineages [17, 18, 23] and the Delta variant that is now dominant might be more than 50% more transmissible than the Alpha variant [20]. If we simply apply these multiplicative terms, *R*_0_ might be as high as 7 for the Delta variant. However, it is possible that transmissibility differences between variants change with control conditions. We therefore considered *R*_0_=5 in our baseline analysis and explored values between 3 and 6 in our sensitivity analyses. For *R*_0_≥5 which appears likely for the Delta variant and under our baseline vaccine coverage of 70–80–90% among teenagers, younger and older adults, we anticipate an important stress on the healthcare system in the absence of any control measure (Fig. 3A). Ongoing efforts to control transmission should therefore be maintained. On a more positive note, thanks to vaccination, the intensity of control measures necessary to maintain hospitalizations at manageable levels should be substantially less than what was required before the roll-out of vaccines. Indeed, while lockdowns used in 2020 reduced transmission rates by 70–80% [10], we find that reductions of the order of 20–30% might now be sufficient. Such reductions might potentially be achieved through protective measures (e.g., masks, hand hygiene), a certain degree of social distancing, teleworking, the sanitary pass, and Test-Trace-Isolate.

Since vaccines reduce the risk of infection and of transmission if infected, our model anticipates that unvaccinated individuals will contribute more to disease spread than vaccinated ones. Since vaccine coverage among children aged 0–17 years old will be low relative to that in adults, we anticipate a strong increase of children’s contribution, with about one third of infections occurring in children and 43% being due to this group in our baseline scenario. Adults that are not vaccinated will also disproportionately contribute to the stress on the healthcare system. This is particularly true for those that are older than 60 years old In our baseline scenario, this group represents 3% of the population but 43% of hospital admissions.

These observations have important implications for epidemic control. First, they show the importance of obtaining near-perfect vaccine coverages in older age groups that contribute disproportionately to the stress on the healthcare system. This likely requires the development of strategies where authorities reach out to individuals to facilitate their access to vaccines. Second, we anticipate that, in a population that is partially vaccinated, gains achieved thanks to social distancing measures are larger when reducing the contacts of unvaccinated individuals rather than those of vaccinated ones. This suggests that, in this new era, control measures targeting unvaccinated individuals (for example with the use of the sanitary pass) may help maximize epidemic control. Such a targeting strategy raises ethical and social issues. From an economic perspective, targeting unvaccinated individuals maximizes the effectiveness of control while minimizing the cost to society. This is consistent with the theory that in situations where a small group of individuals contributes disproportionately to the spread of disease, it is optimal to target that group. However, targeting the unvaccinated leads to forms of discrimination, felt more or less severely. While it is true that discrimination between the vaccinated and unvaccinated is to some extent the result of individual choices, as vaccines are widely available, these choices are nevertheless socially stratified and correlated with age and socioeconomic status. Moreover, the restrictions put in place are not chosen by individuals but defined by the authorities. Choices may therefore be perceived as discrimination, especially by the unvaccinated. in France, while the sanitary pass has been widely accepted, it has also actively mobilized against it minority segments of the population.

Recent data indicate a reduction in vaccine effectiveness against infection by the Delta variant and a waning of immunity against infection, with protection against hospitalization remaining elevated. These changes have important implications for the management of the epidemic since we expect they will facilitate viral circulation even in highly vaccinated populations and eventually increase the stress on the healthcare system. This means that, compared to the first half of 2021, there is a higher risk that vaccinated individuals get infected and transmit the virus. As a consequence, measures reducing the risk of infection and transmission such as the wearing of the mask should apply to vaccinated individuals in situations where transmission is possible (e.g., indoors).

The situation of children is a particular source of concern. Children aged < 12 years old do not have access to vaccines yet and vaccine coverage remains lower among teenagers due to later access to the vaccine. While children mostly develop mild SARS-CoV-2 infections, it is essential to secure their access to education and a normal social life and to protect their mental health. Low vaccine coverage among children puts them at risk of being exposed to class closures, with a deleterious impact on their education and mental health [24]. The vaccination of children would insulate them from that risk. In the case of children, the ethical and social problems are exacerbated. On the vaccination side, discrimination arises from the fact that children cannot be seen as making voluntary choices between vaccination and social restrictions. Vaccination is not yet offered under the age of 12, and beyond the age of 12, the “choice” to be vaccinated depends primarily on the family environment. As for other measures potentially targeted at schools, a wide range of instruments is available (from mask-wearing to physical distancing, air filtration, iterative self-testing, closing rules, dedicated tracing, isolation of family members...) and could help mitigate impact but their targeted implementation would disproportionately affect young people and their families, raising questions of social justice if society at large is less directly targeted, particularly in certain age groups.

This assessment is performed in a context of uncertainty about the value of *R*_0_ and vaccine coverage in the autumn. Our model makes a number of simplifying assumptions. We ignore a potential decay of immunity, whether immunity was acquired through natural infection or vaccination. Our assumption that vaccination reduces the risk of infection by 60% represents an average between the value for individuals that have just been vaccinated (who may have higher protection) and that for those that have been vaccinated some time ago (who may have lower protection) [25]. In the absence of boosting, the decay of immunity might cause the model to be too pessimistic for the start of the Autumn wave and too optimistic for the later part of the wave. It could also mean that the contribution of vaccinated individuals to the epidemic process increases progressively as immunity wanes. We also make the assumption that individuals that have been infected remain protected against reinfection, which may lead to optimistic projections. If the decay of immunity is more important among older individuals, we might expect a larger stress on the healthcare system than anticipated in our baseline scenario since older individuals are more likely to develop severe disease if infected. Integrating the decay of immunity and the impact of booster doses in our model is ongoing and will be the subject of future studies. We consider a national model for France and do not account for spatial heterogeneities, that are important [26]. We considered a 'leaky' vaccine that exhibits failure in degree, as most SARS-CoV-2 models [5, 7, 27, 28]. This assumption could lead to larger epidemic sizes than models with “all-or-nothing” vaccines. For this reason, and given the uncertainty on the basic reproductive ratio for Delta, we performed a sensitivity analysis on *R*_0_.

## Conclusion

We used a mathematical model to anticipate how the epidemiology of SARS-CoV-2 may change in partially vaccinated populations and investigate implications for the control of a possible epidemic rebound this autumn.

## Supplementary Information

**Additional file 1: **Epidemiology and control of SARS-CoV-2 epidemics in partially vaccinated populations: a modeling study applied to France. **Table S1:** Relative risk of infection, transmission and hospitalization for unvaccinated individuals relative to vaccinated individuals, in different age groups. **Figures S1 to S14. Figure S1:** Fit to the data. **Figure S2:** Comparison of the costs of different strategies. **Figure S3:** Distribution of infections between groups defined by their age and vaccination status. **Figure S4:** Distribution of hospitalisations between groups defined by their age and vaccination status. **Figure S5:** Contribution of groups defined by their age and vaccination status to infections, disease spread and hospital burden in a scenario where children aged 0-9 y.o. are 50% less infectious than adults, in addition to being 50% less susceptible. **Figure S6:** Contribution of groups defined by their age and vaccination status to infections, disease spread and hospital burden in a scenario where children aged 0-9 y.o. and teenagers 10-17 are as susceptible as adults. **Figure S7:** Contribution of groups defined by their age and vaccination status to infections, disease spread and hospital burden in a scenario where the efficacy of the vaccines against infection is set to 80%. **Figure S8:** Contribution of groups defined by their age and vaccination status to infections, disease spread and hospital burden in a scenario where the efficacy of the vaccines against hospitalisation is set to 90%. **Figure S9:** Contribution of groups defined by their age and vaccination status to infections, disease spread and hospital burden in a scenario where the vaccinated individuals transmit the virus as the unvaccinated ones. **Figure S10:** Contribution of groups defined by their age and vaccination status to infections, disease spread and hospital burden, in the scenario with R_0_=5 and a vaccine coverage of 80%-90%-90% among 12-17 y.o., 18-59 y.o. and over 60 y.o..**Figure S11:** Contribution of groups defined by their age and vaccination status to infections, disease spread and hospital burden, in the scenario with R0=5 and a vaccine coverage of 80%-90%-90% among 12-17 y.o., 18-59 y.o. and over 60 y.o and where the efficacy of the vaccines against hospitalisation is set to 90%. **Figure S12:** Projections in the absence of control measures, as a function of the basic reproduction number R_0_ and vaccine coverage. **Figure S13:** Projections in the absence of control measures, as a function of the basic reproduction number R_0_ and different vaccine coverages in children aged 5 to 11y.o.. **Figure S14:** Peak in daily hospital admissions under different testing strategies.

## Data Availability

Data and code are available online https://gitlab.pasteur.fr/mmmi-pasteur/sars-cov-2-epidemics-in-partially-vaccinated-populations.

## Abbreviations

SARS-CoV-2: severe acute respiratory syndrome coronavirus 2
VOC: Variant of concern
RR: Risk ratio
