# Supplementary material for: Epidemiology and control of SARS-CoV-2 epidemics in partially vaccinated populations: a modeling study applied to France
Source: BMC Med. 2022 Jan 26;20:33. doi: 10.1186/s12916-022-02235-1 (PMC8789481; doi:10.1186/s12916-022-02235-1)
Supplement: Supplementary file 1 — Additional file 1: Epidemiology and control of SARS-CoV-2 epidemics in partially vaccinated populations: a modeling study applied to France. Table S1: Relative risk of infection, transmission and hospitalization for unvaccinated individuals relative to vaccinated individuals, in different age groups. Figures S1 to S14. Figure S1: Fit to the data. Figure S2: Comparison of the costs of different strategies. Figure S3: Distribution of infections between groups defined by their age and vaccination status. Figure S4: Distribution of hospitalisations between groups defined by their age and vaccination status. Figure S5: Contribution of groups defined by their age and vaccination status to infections, disease spread and hospital burden in a scenario where children aged 0-9 y.o. are 50% less infectious than adults, in addition to being 50% less susceptible. Figure S6: Contribution of groups defined by their age and vaccination status to infections, disease spread and hospital burden in a scenario where children aged 0-9 y.o. and teenagers 10-17 are as susceptible as adults. Figure S7: Contribution of groups defined by their age and vaccination status to infections, disease spread and hospital burden in a scenario where the efficacy of the vaccines against infection is set to 80%. Figure S8: Contribution of groups defined by their age and vaccination status to infections, disease spread and hospital burden in a scenario where the efficacy of the vaccines against hospitalisation is set to 90%. Figure S9: Contribution of groups defined by their age and vaccination status to infections, disease spread and hospital burden in a scenario where the vaccinated individuals transmit the virus as the unvaccinated ones. Figure S10: Contribution of groups defined by their age and vaccination status to infections, disease spread and hospital burden, in the scenario with R0=5 and a vaccine coverage of 80%-90%-90% among 12-17 y.o., 18-59 y.o. and over 60 y.o..Figure S11: Contribution of gro [file 12916_2022_2235_MOESM1_ESM.pdf]

Additional file 1

Epidemiology and control of SARS-CoV-2 epidemics in partially vaccinated populations: a modeling study applied to France

Model parametrization

We developed a deterministic SEEIR model stratified by age similar to the one used in Salje et al. (Salje et al. 2020) The model has been extended to account for the roll-out of vaccines(Tran Kiem et al. 2021) as well as the deployment of self-administered rapid antigenic tests(Bosetti et al. 2021). The metropolitan French population is divided into the following 13 age groups: [0-10), [10-18), [18-30), [30-40), [40-45), [45-50), [50-55), [55-60), [60-65), [65-70), [70-75), [75-80) and ≥ 80. We assume that individuals aged 0-9 y.o. and 10-17 y.o are respectively 50% and 25% less susceptible compared to adults(Viner et al. 2021b; Davies et al. 2020). The model is implemented with the R software using the *odin* package(FitzJohn 2020).

Transmission model accounting for random testing

Upon infection, susceptible individuals (S) move to the compartment E1. After an average duration of 4.0 days, infected individuals move to the E2 compartment where they become infectious. They stay in this compartment for an average duration of 1.0 day before moving to the I compartment (IM for mild infections or IH for infections requiring an admission in hospital) in which a fraction of them will develop symptoms. The average length of stay in I is equal to 3.0 days. The proportion of infected people who will need to be admitted to the hospital is age-dependent and accounts for the increased severity associated with the Delta variant. Specifically, Delta VOC is assumed to be 50% more severe than Alpha VOC(Twohig et al. 2021), whereas Alpha VOC is assumed to be 40% more severe than historical strains(Bager et al., n.d.). The age-dependent probability of hospitalization for the historical strains is obtained from Lapidus et al(Lapidus et al. 2021). Finally, individuals in the IM compartment will recover (R compartment), while individuals in the IH will move to the $\bar{I}H$ compartment before being admitted in hospital (compartment H). Individuals who have been vaccinated follow the same path as those who have not been vaccinated, but they are less susceptible to infection, have a reduced risk of being hospitalized, and are less likely to transmit the disease (Tran Kiem et al. 2021).

Our framework accounts for the deployment of random testing strategies. Upon receiving a positive test, we assume that infectious individuals (in compartments E2, IM, and IH) detected isolate, resulting in a reduction of their transmission rate by 75%. This corresponds to the compartments E2iso, IMiso, and IHiso. We assume that individuals tested while in E1 remain undetected and therefore do not isolate. The average length of stay in isolated compartments is identical to the one in non-isolated ones.

Accounting for age-specific susceptibility and infectivity

Children and teenagers have been suggested to be less susceptible to SARS-CoV-2 infection upon contact with an infectious individual compared to adults (Davies et al. 2020; Viner et al. 2021a). In our reference scenario, we assume that children aged 0-9 y.o.

(respectively 10-17 y.o.) are 50% (respectively 25%) less susceptible than adults aged 18 y.o. and older. In a sensitivity analysis, we explore a scenario where children aged 0-9 y.o. are additionally 50% less infectious than individuals aged 10 y.o. and older (Li et al. 2021). We account for age-varying susceptibilities and infectivities by computing an adjusted mixing matrix $C = (C_{i,j})$ from the contact matrix $C^{CS} = (C^{CS}_{i,j})$ derived from a French contact survey (Béraud et al. 2015). Let σ_i denote the relative susceptibility of age-group i compared to adults aged 18 y.o. and older and θ_i denote the relative infectivity of individuals within age group i compared to adults aged 18 y.o. and older. The coefficients of the adjusted mixing matrix are derived as:

$$C_{i,j} = \sigma_i \cdot C^{CS}_{i,j} \cdot \theta_j$$

Model Equations

The model can be described by the following set of ordinary differential equations:

$$dS_i/dt = -S_i \beta(\Sigma_j C_{ij}((1-\rho_{int})(E2_j + I_j^{mild} + I_j^{hosp} + (1-\rho_{iso})(E2iso_j + Iiso_j^{mild} + Iiso_j^{hosp}))$$
$$+ (1-\rho^v_{int})(1-VE_{inf})(E2^v_j + I^{v\,mild}_j + I^{v\,hosp}_j + (1-\rho_{iso})(E2iso^v_j + Iiso^{v\,mild}_j + Iiso^{v\,hosp}_j)))/N_j)$$

$$dS^v_i/dt =$$
$$-(1 - VE_{susc}) S^v_i \beta(\Sigma_j C_{ij}((1-\rho_{int})(E2_j + I_j^{mild} + I_j^{hosp} + (1-\rho_{iso})(E2iso_j + Iiso_j^{mild} + Iiso_j^{hosp}))$$
$$+ (1-\rho^v_{int})(1-VE_{inf})(E2^v_j + I^{v\,mild}_j + I^{v\,hosp}_j + (1-\rho_{iso})(E2iso^v_j + Iiso^{v\,mild}_j + Iiso^{v\,hosp}_j)))/N_j)$$

$$dE1_i/dt = S_i \beta(\Sigma_j C_{ij}((1-\rho_{int})(E2_j + I_j^{mild} + I_j^{hosp} + (1-\rho_{iso})(E2iso_j + Iiso_j^{mild} + Iiso_j^{hosp}))$$
$$+ (1-\rho^v_{int})(1-VE_{inf})(E2^v_j + I^{v\,mild}_j + I^{v\,hosp}_j + (1-\rho_{iso})(E2iso^v_j + Iiso^{v\,mild}_j + Iiso^{v\,hosp}_j)))/N_j)$$
$$- g_1 \cdot E1_i$$

$$dE1^v_i/dt =$$
$$(1 - VE_{susc}) S^v_i \beta(\Sigma_j C_{ij}((1-\rho_{int})(E2_j + I_j^{mild} + I_j^{hosp} + (1-\rho_{iso})(E2iso_j + Iiso_j^{mild} + Iiso_j^{hosp}))$$
$$+ (1-\rho^v_{int})(1-VE_{inf})(E2^v_j + I^{v\,mild}_j + I^{v\,hosp}_j + (1-\rho_{iso})(E2iso^v_j + Iiso^{v\,mild}_j + Iiso^{v\,hosp}_j)))/N_j)$$
$$- g_1 \cdot E1^v_i$$

$$dE2_i/dt \;=\; g_1 \cdot E1_i - g_2 \cdot E2_i - \nu_{test,i} \cdot E2_i$$

$$dE2^v_i/dt \;=\; g_1 \cdot E1^v_i - g_2 \cdot E2^v_i - v^v_{test,i} \cdot E2^v_i$$

$$dE2iso_i/dt \;=\; \nu_{test,i} \cdot E2_i - g_2 \cdot E2iso_i$$

$$dE2iso^v_i/dt \;=\; v^v_{test,i} \cdot E2^v_i - g_2 \cdot E2iso^v_i$$

$$dI_i^{mild}/dt \;=\; (1 - p^{hosp}_i) \cdot g_2 \cdot E2_i - g_3 \cdot I_i^{mild} - v_{test,i} \cdot I_i^{mild}$$

$$dI^{v\;mild}_i/dt \;=\; (1 - p^{hosp}_i \cdot (1 - VE_{sev}))\, g_2 \cdot E2^v_i - g_3 \cdot I^{v\;mild}_i - v^v_{test,i} \cdot I^{v\;mild}_i$$

$$dIiso_i^{mild}/dt \;=\; (1 - p^{hosp}_i) \cdot g_2 \cdot E2iso_i + v_{test,i} \cdot I_i^{mild} - g_3 \cdot Iiso_i^{mild}$$

$$dIiso^{v\;mild}_i/dt \;=\; (1 - p^{hosp}_i \cdot (1 - VE_{sev}))\, g_2 \cdot E2iso^v_i + v^v_{test,i} \cdot I^{v\;mild}_i - g_3 \cdot Iiso^{v\;mild}_i$$

$$dR_i/dt \;=\; g_3 \cdot I_i^{mild} + g_3 \cdot Iiso_i^{mild}$$

$$dR^v_i/dt \;=\; g_3 \cdot I^{v\;mild}_i + g_3 \cdot Iiso^{v\;mild}_i$$

$$dI_i^{hosp}/dt \;=\; p^{hosp}_i \cdot g_2 \cdot E2_i - g_3 \cdot I_i^{hosp} - v_{test,i} \cdot I_i^{hosp}$$

$$dI^{v\;hosp}_i/dt \;=\; p^{hosp}_i \cdot (1 - VE_{sev}) \cdot g_2 \cdot E2^v_i - g_3 \cdot I^{v\;hosp}_i - v^v_{test,i} \cdot I^{v\;hosp}_i$$

$$dIiso_i^{hosp}/dt \;=\; p^{hosp}_i \cdot g_2 \cdot E2iso_i + v_{test,i} \cdot I_i^{hosp} - g_3 \cdot Iiso_i^{hosp}$$

$$dIiso^{v\;hosp}_i/dt \;=\; p^{hosp}_i \cdot (1 - VE_{sev}) \cdot g_2 \cdot E2iso^v_i + v^v_{test,i} \cdot I^{v\;hosp}_i - g_3 \cdot Iiso^{v\;hosp}_i$$

$$d\bar{\mathrm{I}}H_i/dt \;=\; g_3 \cdot I_i^{hosp} + g_3 \cdot Iiso_i^{hosp} - g_4 \cdot \bar{\mathrm{I}}H_i$$

$$d\bar{I}H^v_i/dt = g_3 \cdot I^{v\ hosp}_i + g_3 \cdot Iiso^{v\ hosp}_i - g_4 \cdot \bar{I}H^v_i$$

$$dH_i/dt = g_4 \cdot \bar{I}H_i$$

$$dH^v_i/dt = g_4 \cdot \bar{I}H^v_i$$

where we let:

- C_{ij}, $(i, j) \in \{1, ..., 13\}^2$ denote the coefficient of the contact matrix,
- the superscripts v indicate the different vaccinated compartments,
- the subscripts i indicate the age groups,
- N_j denote the population size for the age class j,
- β denote the transmission rate,
- g_1 denote the rate at which an exposed individual becomes infectious and we set its value $1/g_1 = 4$ days. We set $1/g_2 = 1$ day, and $1/g_3 = 3$days resulting in an average infectious period of 4 days,
- $g_4$denote the rate of hospital admissions and we set $1/g_4 = 4$days,(Salje et al. 2020)
- ρ_{int} denote the reduction in the transmission rate for unvaccinated individuals (impact of non-pharmaceutical interventions),
- ρ^v_{int} denote the reduction in the transmission rate for vaccinated individuals (impact of non-pharmaceutical interventions),
- ρ_{iso}denote the reduction in the transmission rate for isolated individuals,
- $\nu_{test,i}$denote the rate of testing for unvaccinated individuals.
- $\nu^v_{test,i}$denote the rate of testing for vaccinated individuals,
- VE_{sev}, VE_{inf}, and VE_{susc} denote the effectiveness of the vaccines on reducing the probability of hospitalization, the infectiousness and the probability of becoming infected of vaccinated individuals compared to unvaccinated individuals.

Random testing

Let assume p^{test}_i the proportion of the population of the age class i participating in random testing. In the scenario where only unvaccinated individuals aged ≥12 y.o. take part in testing, we set

$$\nu_{test,i} = p^{test}_i \cdot Sensitivity \cdot (1/test_{delay}) \text{ and}$$

$$\nu^v_{test,i} = 0$$

where $Sensitivity$ denotes the test sensitivity (equal to 75% if the test is self administered and or 90% if it is performed by a professional), and $test_{delay}$ represents the number of days between two consecutive tests (7 days in the baseline scenario).
In the scenario where individuals participating in the testing campaign are drawn randomly in the population aged ≥12 y.o. (vaccinated and unvaccinated) we set

$$\nu_{test,i} = \nu^{v}_{test,i} = p^{test}_{i} \cdot p_{unvaccinated,i} \cdot Sensitivity \cdot (1/test_{delay}),$$

where $p_{unvaccinated,i}$ represent the proportion of unvaccinated individuals of the age class i. This ensures that the daily number of individuals tested in the strategy targeting the unvaccinated population is equal to the daily number of individuals tested in the untargeted strategy.

Initialization of the model on September 1st, 2021

To account for heterogeneity in the risk of infection between the different age groups of the population, we use the distribution of infections predicted by our dynamical model calibrated on data until May 15th 2021 (Figure S1). On that date, we estimate that about 20% of the population in metropolitan France was infected by SARS-CoV-2. During summer 2021, the metropolitan France experienced a wave of infection due to the arrival of the Delta variant. So, on September 1st, 2021, we assume that 25% (range: 20%-30%) of the metropolitan French population developed immunity through natural infection. The natural infections are thus distributed across different age groups to reproduce both the distribution of infections obtained from the model and the proportion of the population having acquired immunity. We also build several scenarios regarding the vaccine coverages reached in different groups of the population:

- 90% or 95% among those older than 60 years old (y.o.)
- 60%, 80% or 90% among those aged 18-59 y.o.
- 0%, 30% or 70% among the 12-17 y.o.

In our baseline scenario we assume a vaccination coverage of 70%, 80% and 90% among 12-17 y.o., 18-59 y.o. and over 60 y.o. respectively on September 1st, 2021.

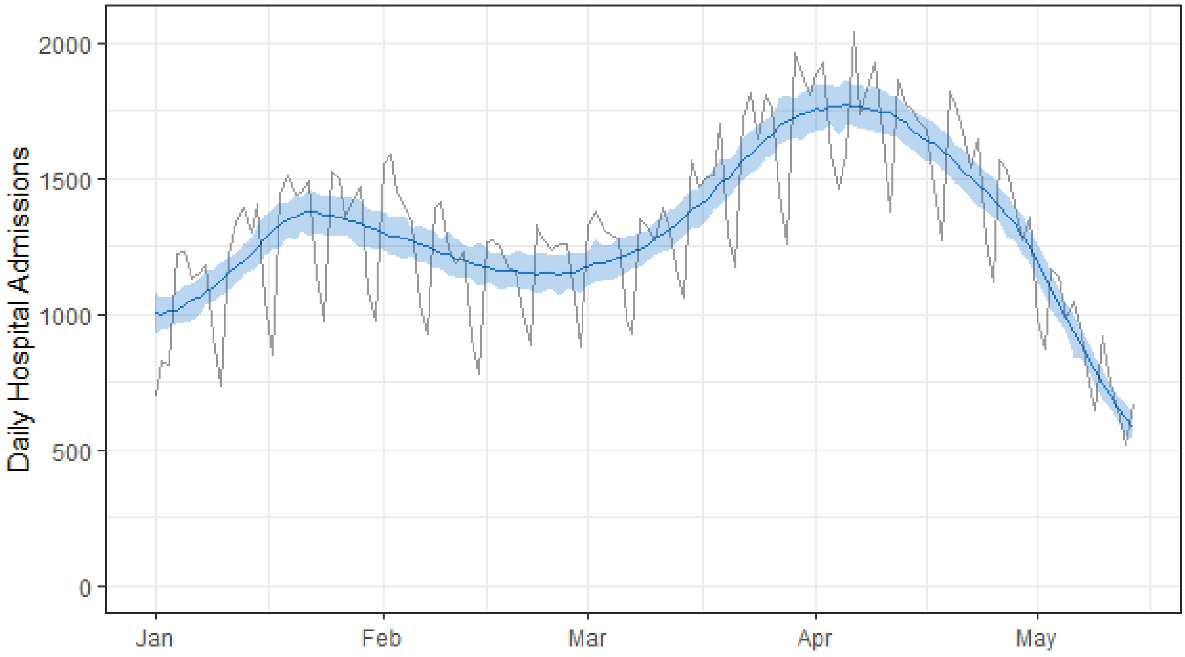

Figure S1: Fit to the data. Daily hospital admissions through time. The blue line and area correspond to model posterior mean and 95% credible intervals, while the grey line corresponds to data.

Additional results

Table S1: Relative risk of infection, transmission and hospitalization for unvaccinated individuals relative to vaccinated individuals, in different age groups. This is for our baseline scenario characterized by R_0=5 and a vaccine coverage of 70%-80%-90% among 12-17 y.o., 18-59 y.o. and over 60 y.o.

**Age group**	**Infection**	**Transmission**	**Hospitalisation**
**0-17**	1.3	2.2	18.9
**18-59**	1.9	3.8	15.0
**60+**	2.2	4.3	17.3
**All**	2.1	4.3	6.1

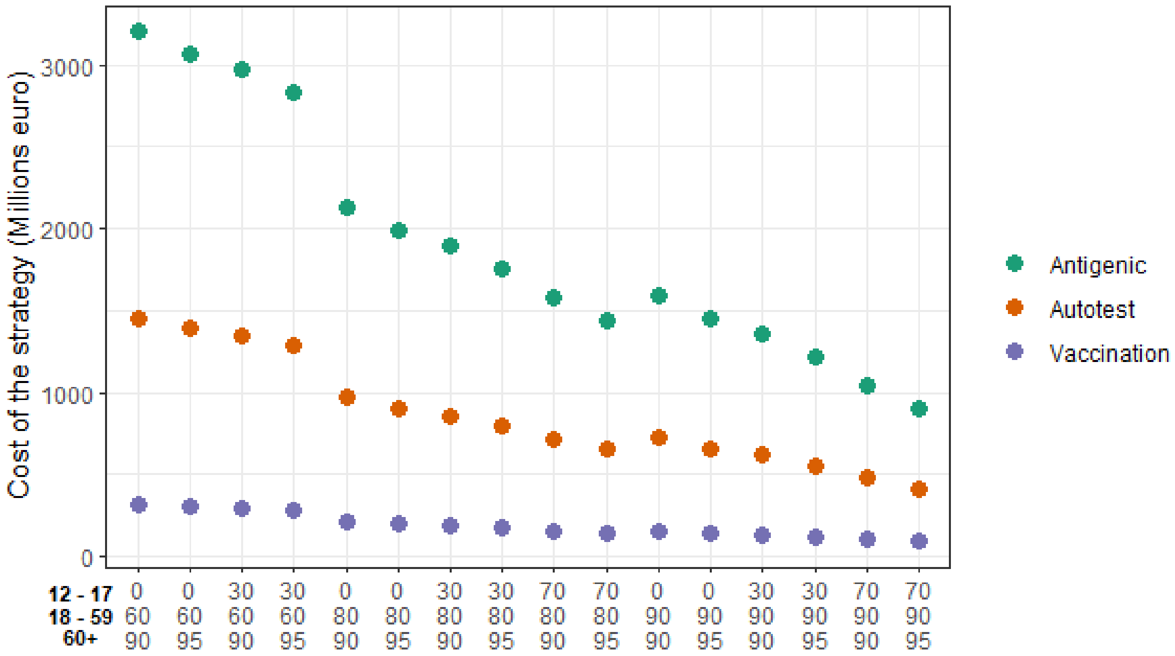

Figure S2: Comparison of the costs of different strategies. Costs of strategies targeting 50% of the unvaccinated individuals older than 12 y.o. as a function of the vaccine coverage reached in different groups. The 3 strategies are: weekly testing with an antigenic test performed by a professional ("antigenic"), weekly testing with an antigenic test performed by the individual ("autotest"), vaccination.

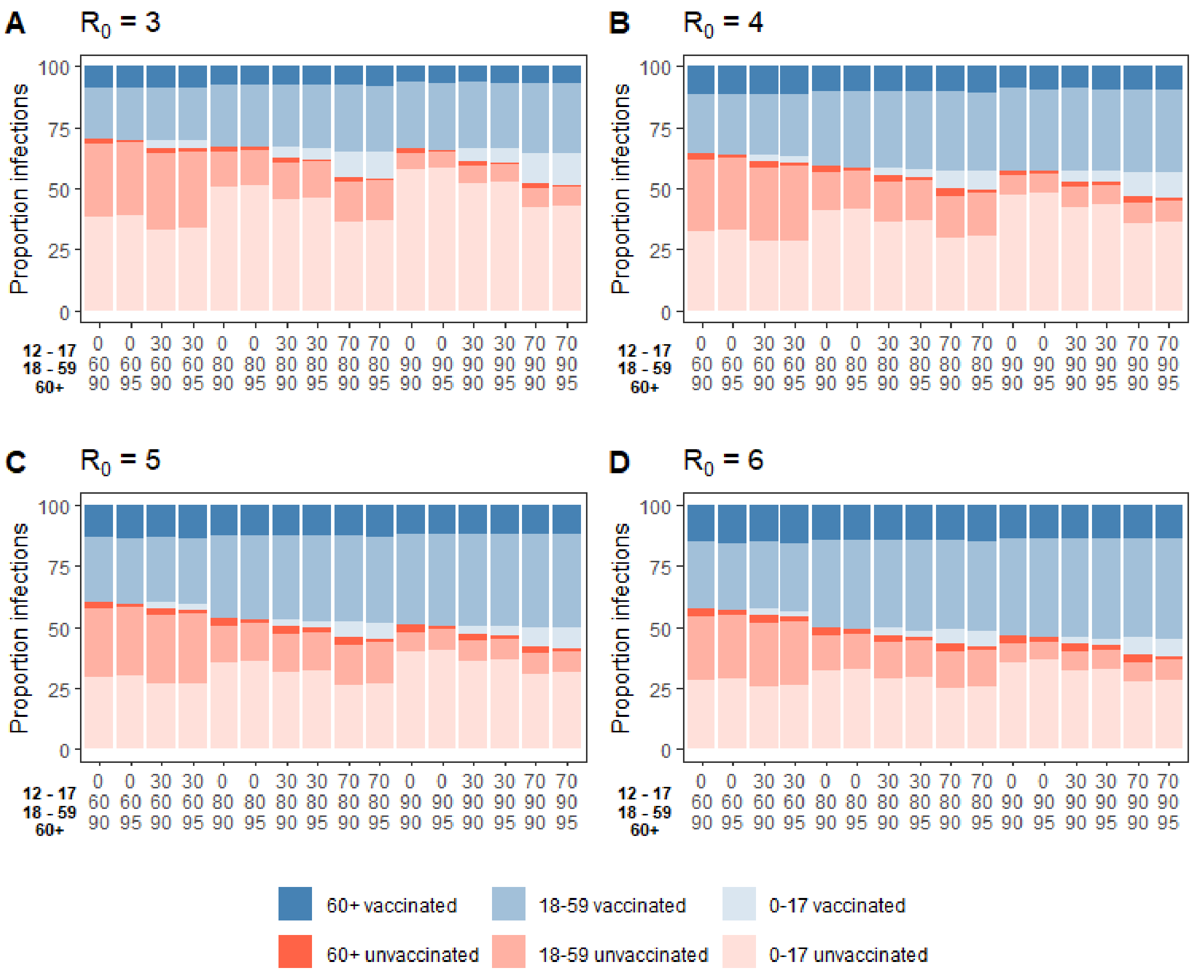

Figure S3: Distribution of infections between groups defined by their age and vaccination status. A. for R_0 = 3. **B.** for R_0 = 4. **C.** for R_0 = 5. **D.** for R_0 = 6. The distribution is reported for infections occurring between September 1st, 2021 and March 20th, 2022 (end of the study period) and as a function of the vaccine coverage reached in the 12-17 y.o., 18-59 y.o. and over 60 y.o.

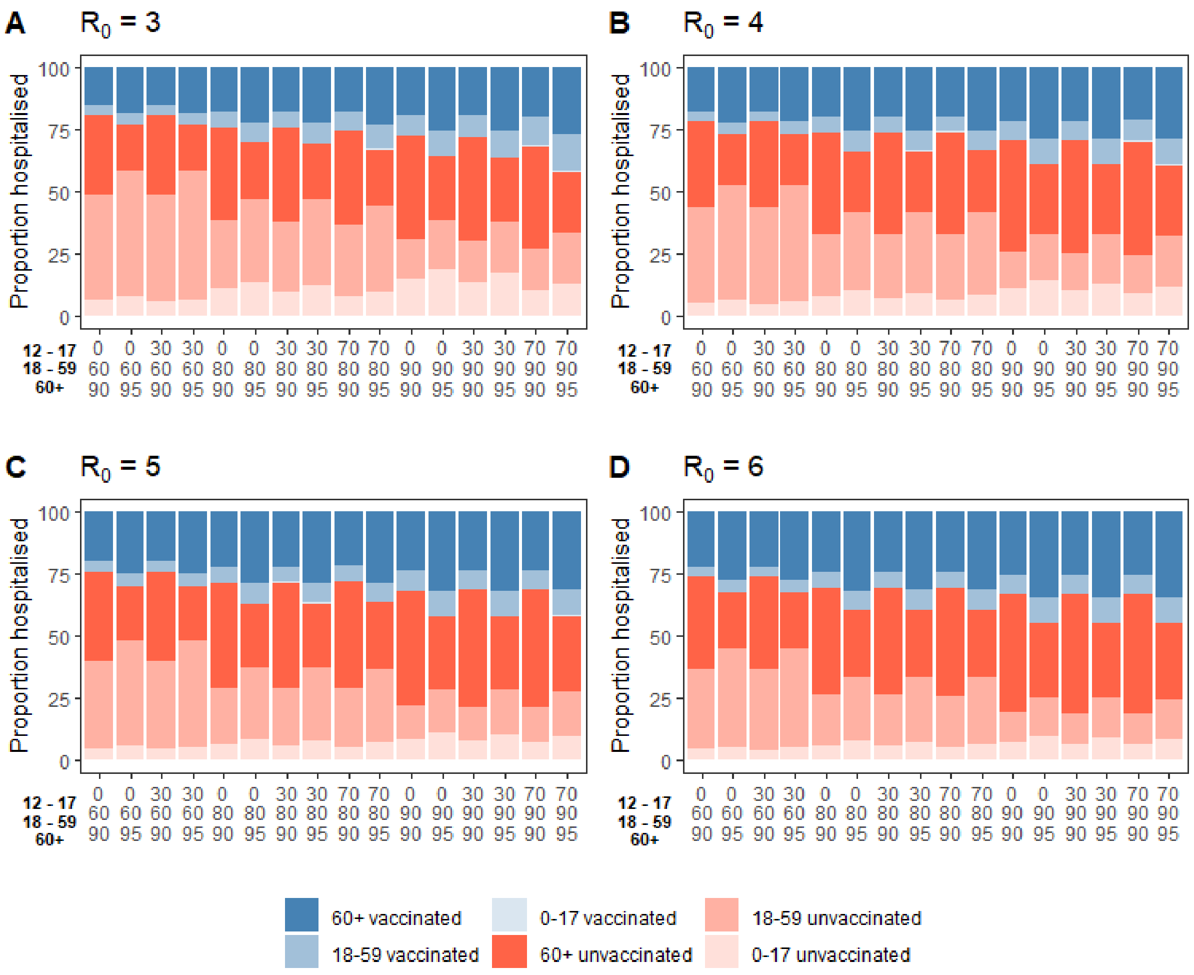

Figure S4: Distribution of hospitalisations between groups defined by their age and vaccination status. A. for R_0 = 3. **B.** for R_0 = 4. **C.** for R_0 = 5. **D.** for R_0 = 6. The distribution is reported for hospitalizations occurring between September 1st, 2021 and March 20th, 2022 (end of the study period) and as a function of the vaccine coverage reached in the 12-17 y.o., 18-59 y.o. and over 60 y.o.

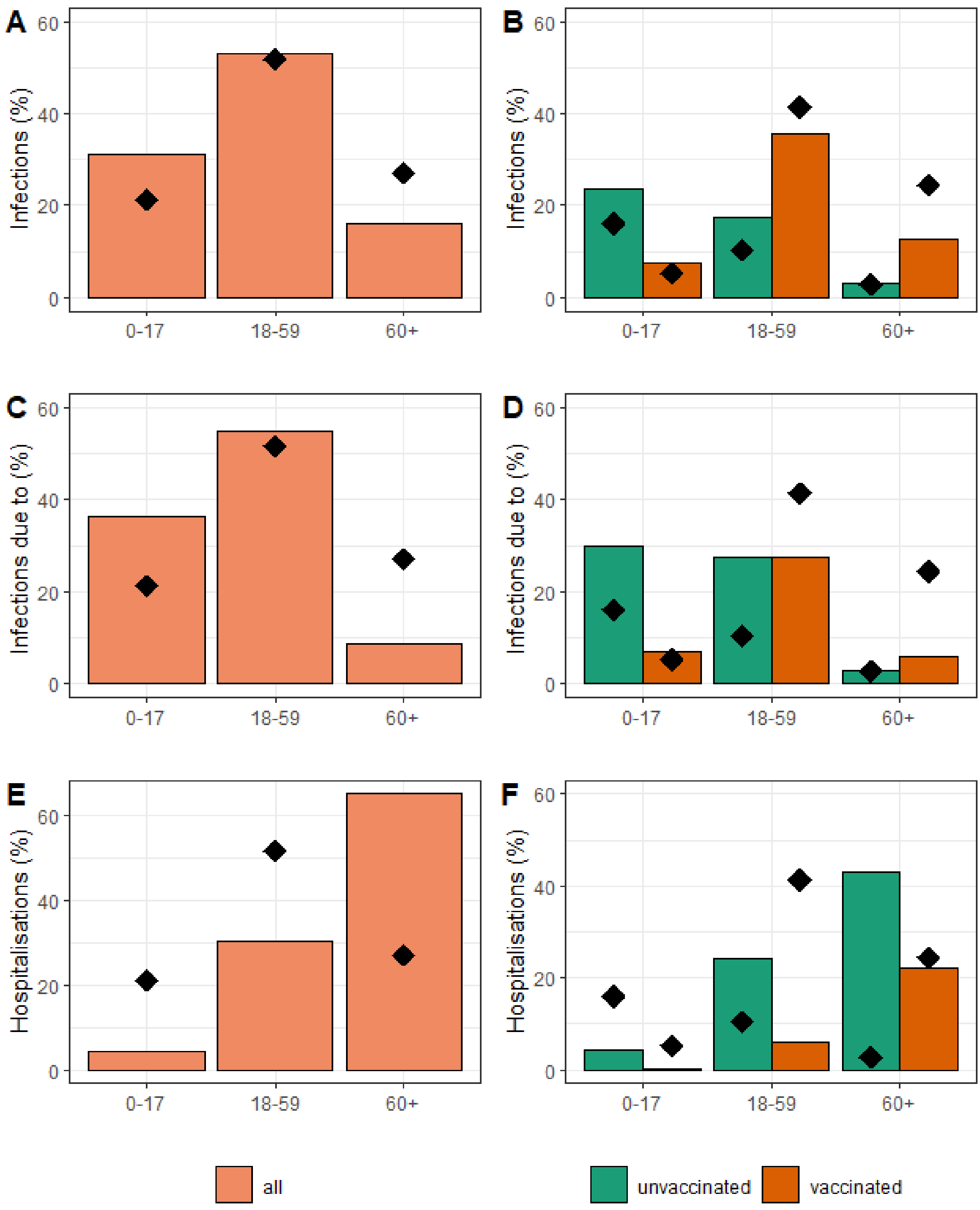

Figure S5: Contribution of groups defined by their age and vaccination status to infections, disease spread and hospital burden in a scenario where children aged 0-9 y.o. are 50% less infectious than adults, in addition to being 50% less susceptible. This is done under our baseline assumptions with R_0=5 and a vaccine coverage of 70%-80%-90% among 12-17 y.o., 18-59 y.o. and over 60 y.o. Age distribution of new infections **A.** in the entire population and **B.** among vaccinated and unvaccinated individuals. Proportion of infections **C.** attributable to different age groups and **D.** attributable to different age groups among vaccinated and unvaccinated individuals. Age distribution of

hospitalisations **E.** in the entire population and **F.** among vaccinated and unvaccinated individuals. In all panels, the diamonds indicate the age distribution of the different groups in the population.

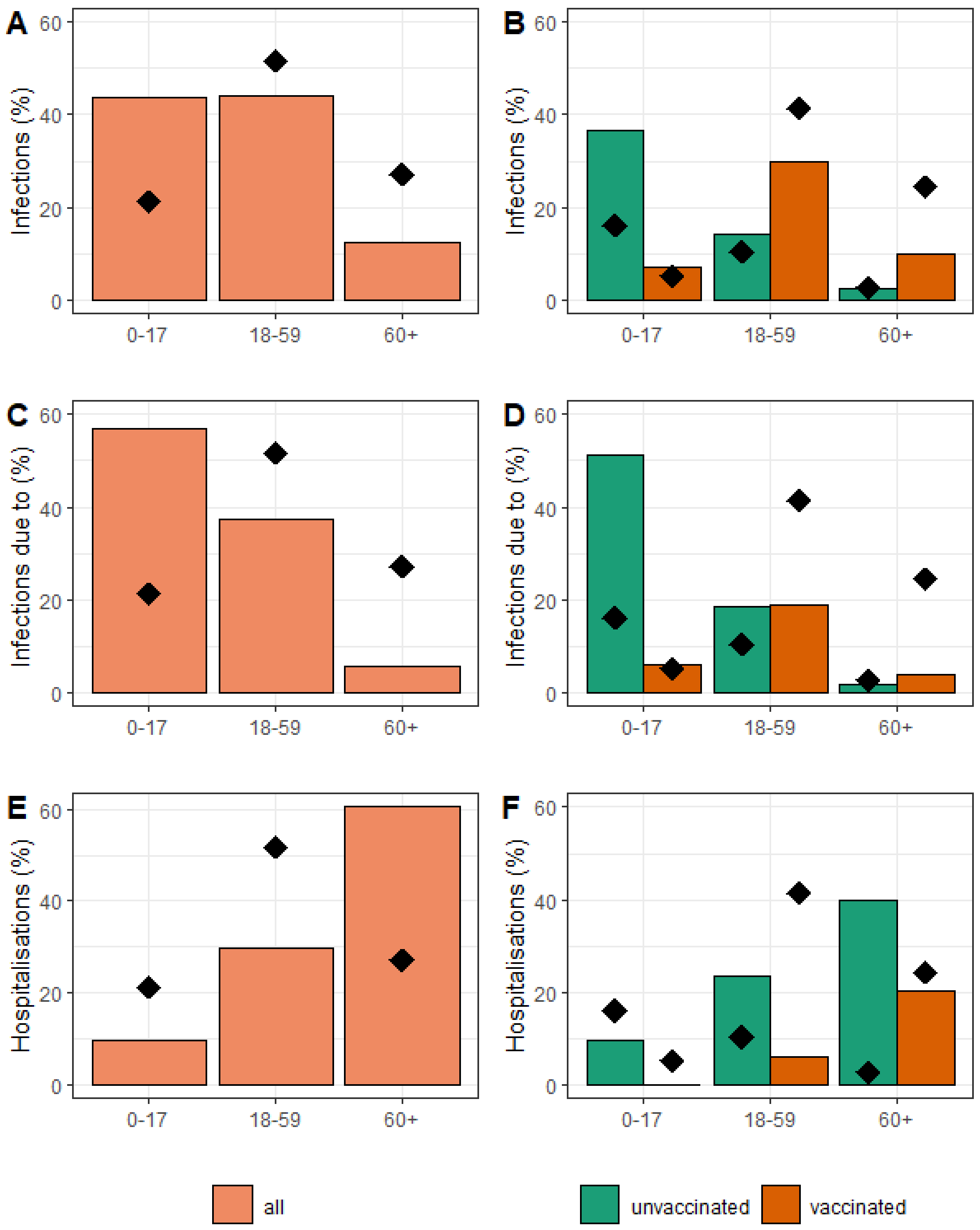

Figure S6: Contribution of groups defined by their age and vaccination status to infections, disease spread and hospital burden in a scenario where children aged 0-9 y.o. and teenagers 10-17 are as susceptible as adults. This is done under our baseline assumptions with R_0=5 and a vaccine coverage of 70%-80%-90% among 12-17 y.o., 18-59 y.o. and over 60 y.o. Age distribution of new infections **A.** in the entire population and **B.** among vaccinated and unvaccinated individuals. Proportion of infections **C.** attributable to different age groups and **D.** attributable to different age groups among vaccinated and unvaccinated individuals. Age distribution of hospitalisations **E.** in the entire population and

F. among vaccinated and unvaccinated individuals. In all panels, the diamonds indicate the age distribution of the different groups in the population.

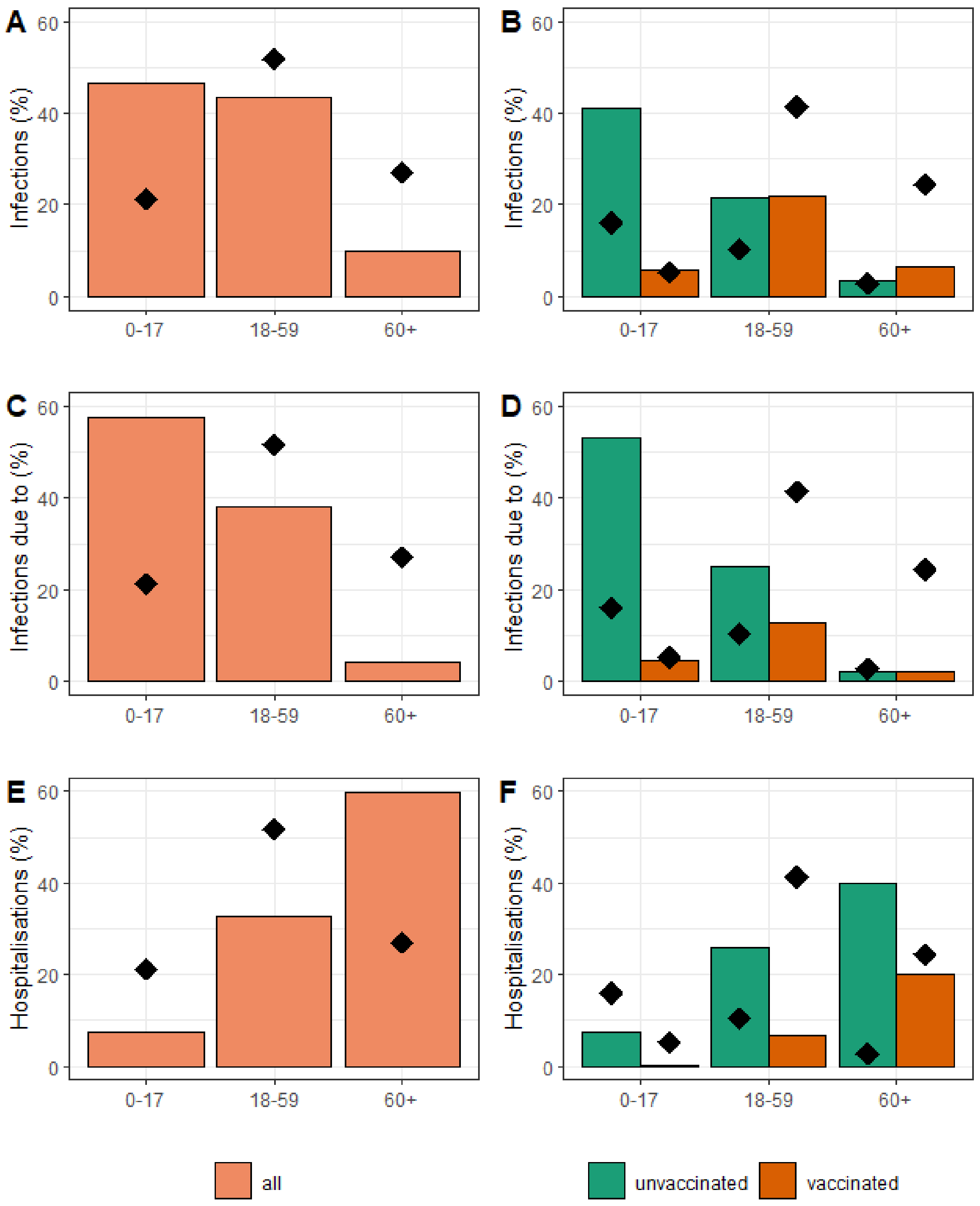

Figure S7: Contribution of groups defined by their age and vaccination status to infections, disease spread and hospital burden in a scenario where the efficacy of the vaccines against infection is set to 80%. This is done under our baseline assumptions with R_0=5 and a vaccine coverage of 70%-80%-90% among 12-17 y.o., 18-59 y.o. and over 60 y.o. Age distribution of new infections **A.** in the entire population and **B.** among vaccinated and unvaccinated individuals. Proportion of infections **C.** attributable to different age groups and **D.** attributable to different age groups among vaccinated and unvaccinated individuals. Age distribution of hospitalisations **E.** in the entire population and **F.** among

vaccinated and unvaccinated individuals. In all panels, the diamonds indicate the age distribution of the different groups in the population.

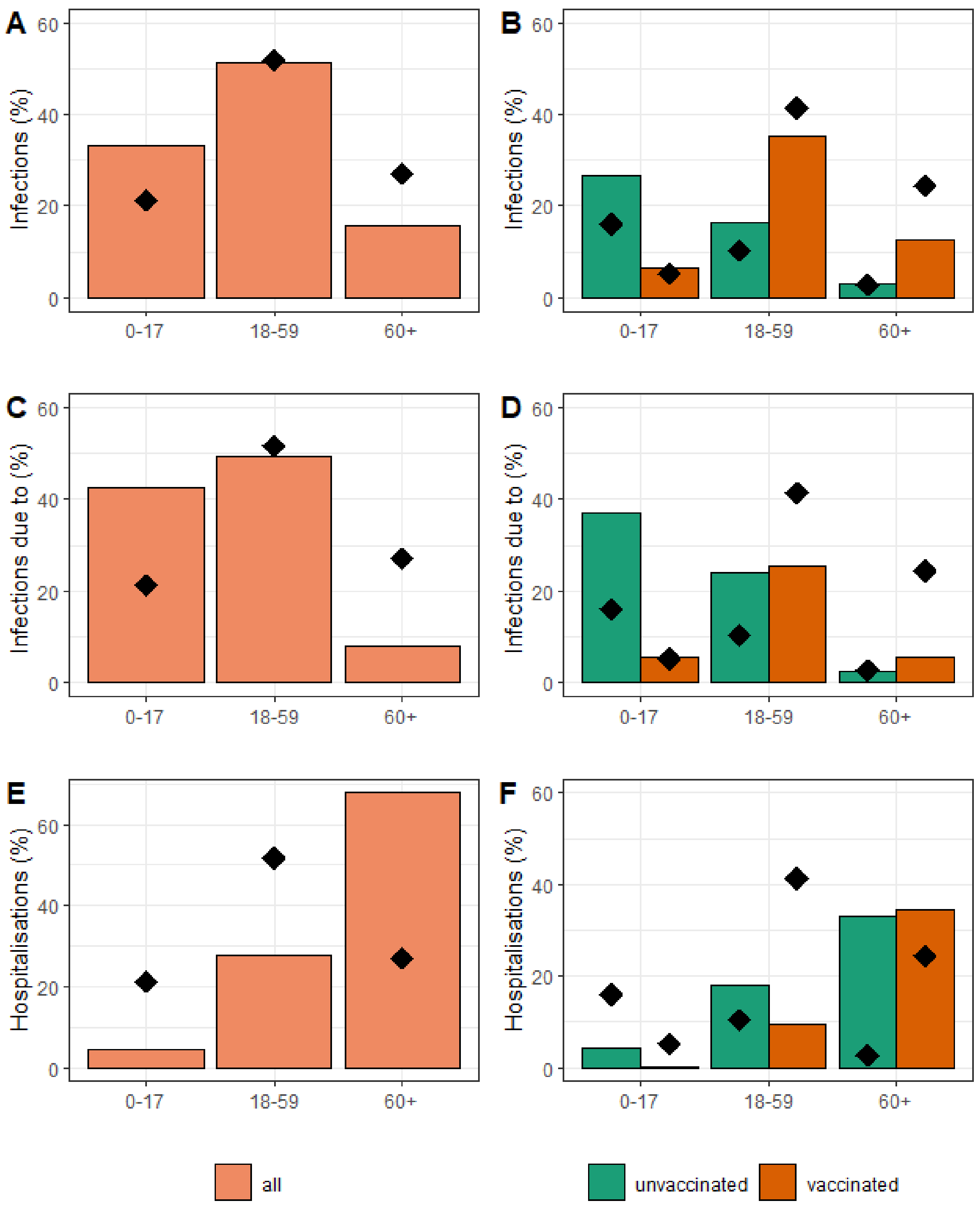

Figure S8: Contribution of groups defined by their age and vaccination status to infections, disease spread and hospital burden in a scenario where the efficacy of the vaccines against hospitalisation is set to 90%. This is done under our baseline assumptions with R_0=5 and a vaccine coverage of 70%-80%-90% among 12-17 y.o., 18-59 y.o. and over 60 y.o. Age distribution of new infections **A.** in the entire population and **B.** among vaccinated and unvaccinated individuals. Proportion of infections **C.** attributable to different age groups and **D.** attributable to different age groups among vaccinated and

unvaccinated individuals. Age distribution of hospitalisations **E.** in the entire population and **F.** among vaccinated and unvaccinated individuals. In all panels, the diamonds indicate the age distribution of the different groups in the population.

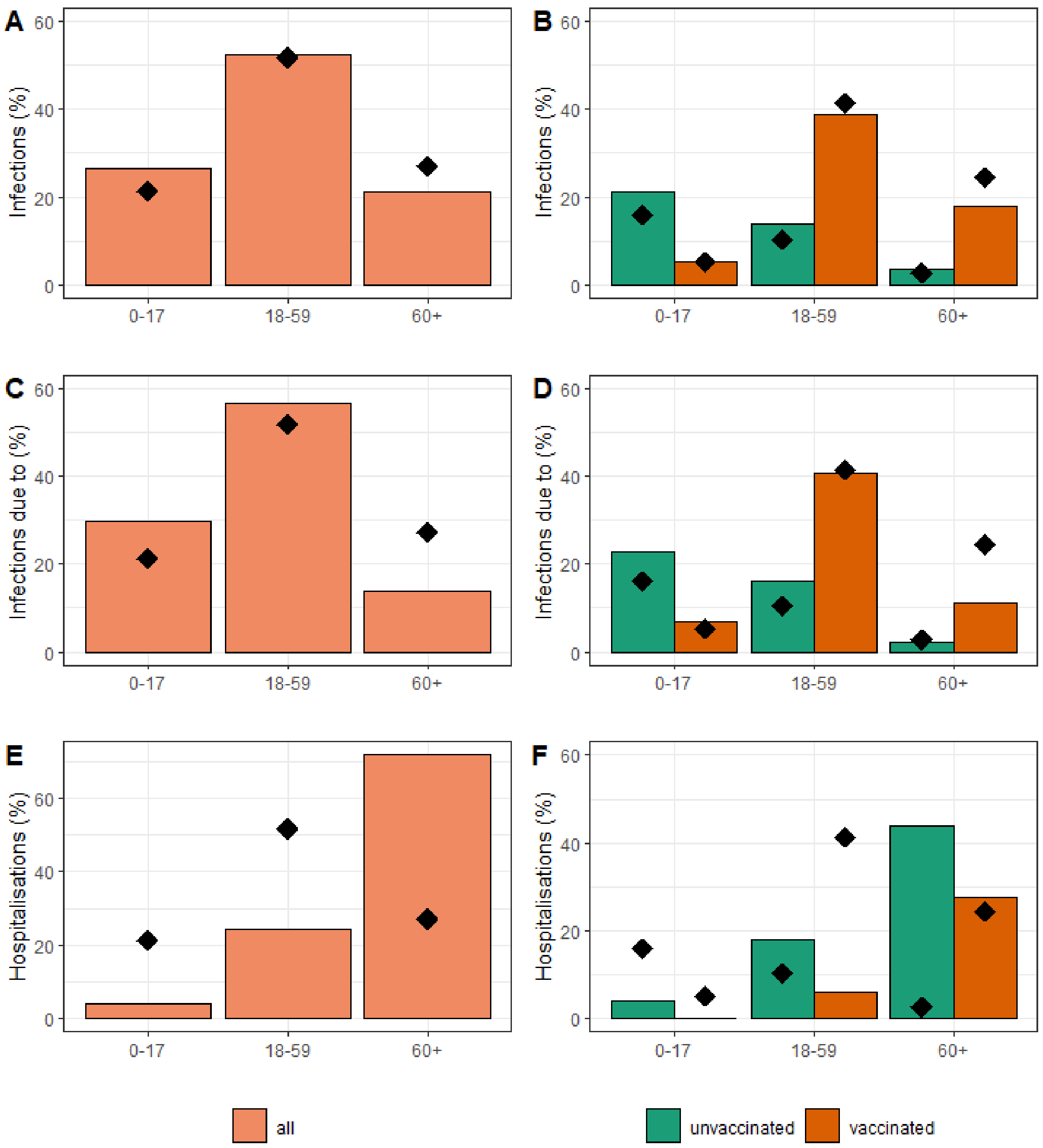

Figure S9: Contribution of groups defined by their age and vaccination status to infections, disease spread and hospital burden in a scenario where the vaccinated individuals transmit the virus as the unvaccinated ones. This is done under our baseline assumptions with R_0=5 and a vaccine coverage of 70%-80%-90% among 12-17 y.o., 18-59 y.o. and over 60 y.o. Age distribution of new infections **A.** in the entire population and **B.** among vaccinated and unvaccinated individuals. Proportion of infections **C.** attributable to different age groups and **D.** attributable to different age groups among vaccinated and unvaccinated individuals. Age distribution of hospitalisations **E.** in the entire population and **F.** among vaccinated and unvaccinated individuals. In all panels, the diamonds indicate the age distribution of the different groups in the population.

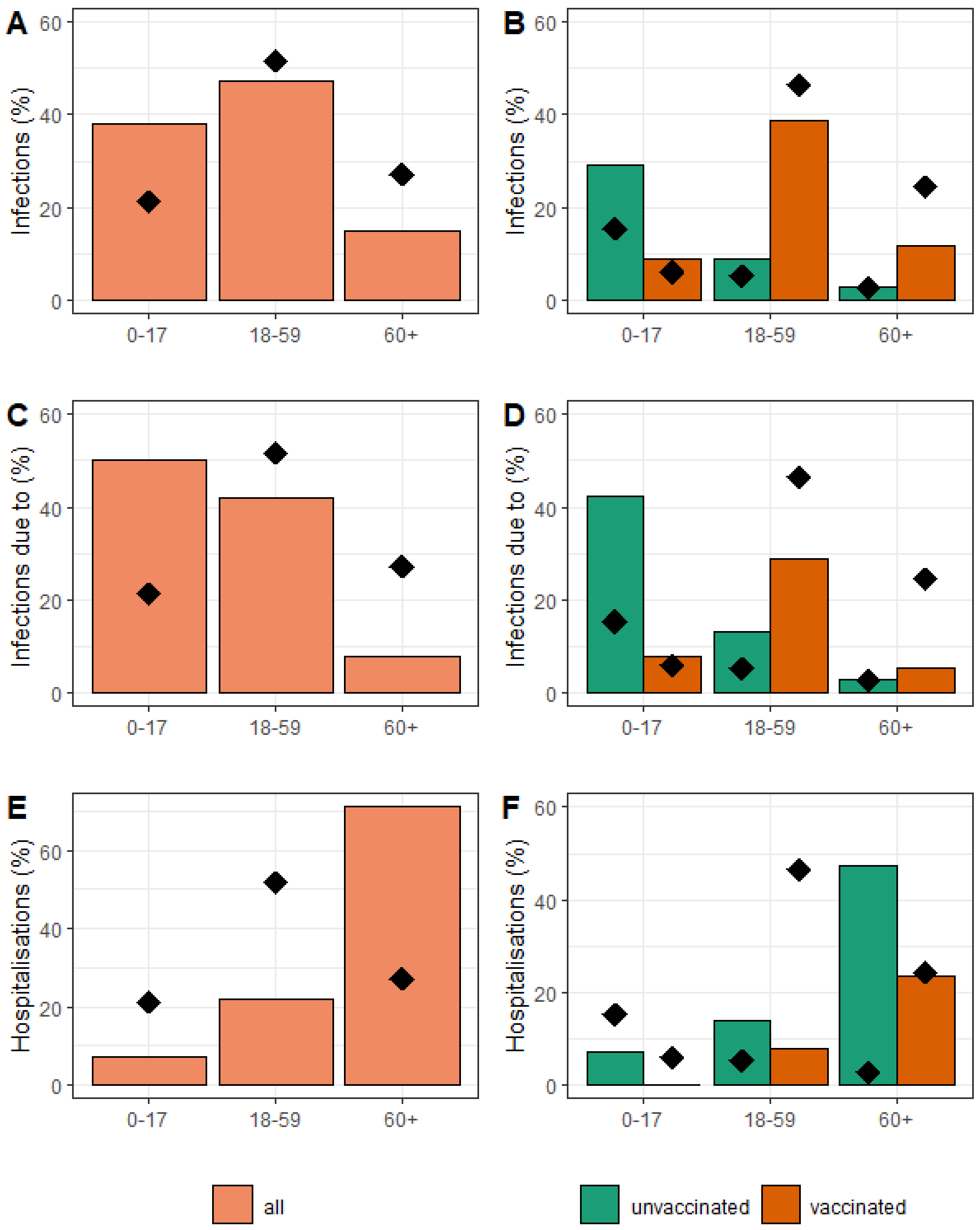

Figure S10: Contribution of groups defined by their age and vaccination status to infections, disease spread and hospital burden, in the scenario with R_0=5 and a vaccine coverage of 80%-90%-90% among 12-17 y.o., 18-59 y.o. and over 60 y.o. Age distribution of new infections **A.** in the entire population and **B.** among vaccinated and unvaccinated individuals. Proportion of infections **C.** attributable to different age groups and **D.** attributable to different age groups among vaccinated and unvaccinated individuals. Age distribution of hospitalisations **E.** in the entire population and **F.** among vaccinated and

unvaccinated individuals. In all panels, the diamonds indicate the age distribution of the different groups in the population.

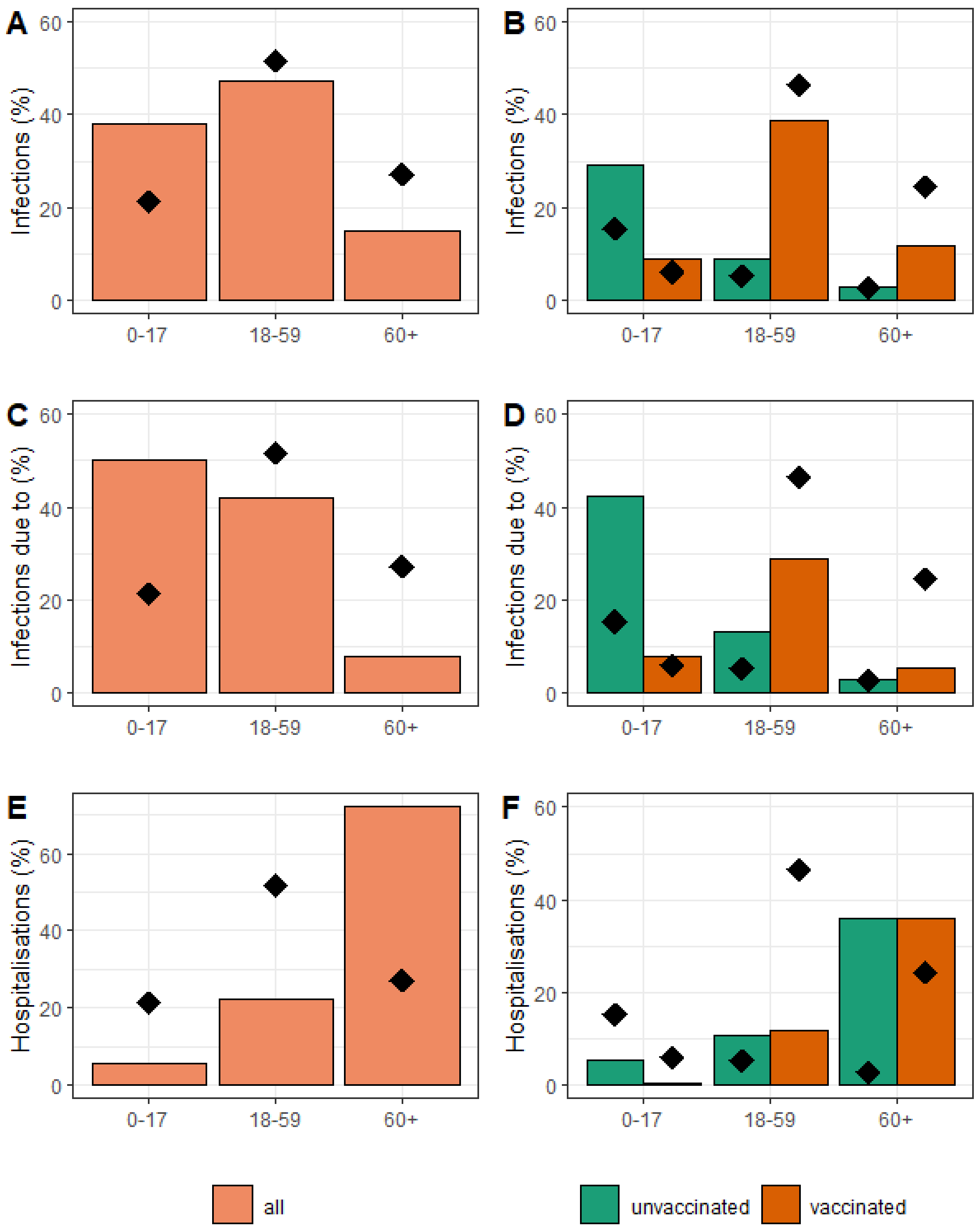

Figure S11: Contribution of groups defined by their age and vaccination status to infections, disease spread and hospital burden, in the scenario with R0=5 and a vaccine coverage of 80%-90%-90% among 12-17 y.o., 18-59 y.o. and over 60 y.o and where the efficacy of the vaccines against hospitalisation is set to 90%. Age distribution of new infections A. in the entire population and B. among vaccinated and unvaccinated individuals. Proportion of infections C. attributable to different age groups and D. attributable to different age groups among vaccinated and unvaccinated individuals. Age distribution of hospitalisations E. in the entire population and F. among vaccinated and

unvaccinated individuals. In all panels, the diamonds indicate the age distribution of the different groups in the population.

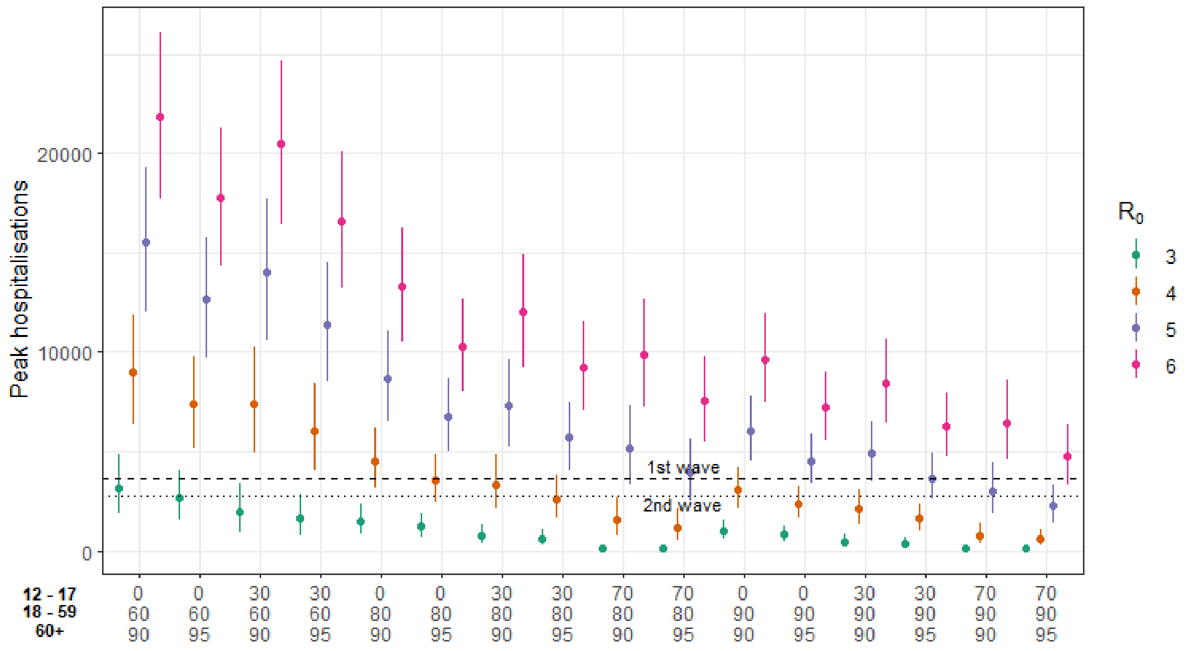

Figure S12: Projections in the absence of control measures, as a function of the basic reproduction number R_0 and vaccine coverage. Peak in daily hospital admissions in the absence of control measures.

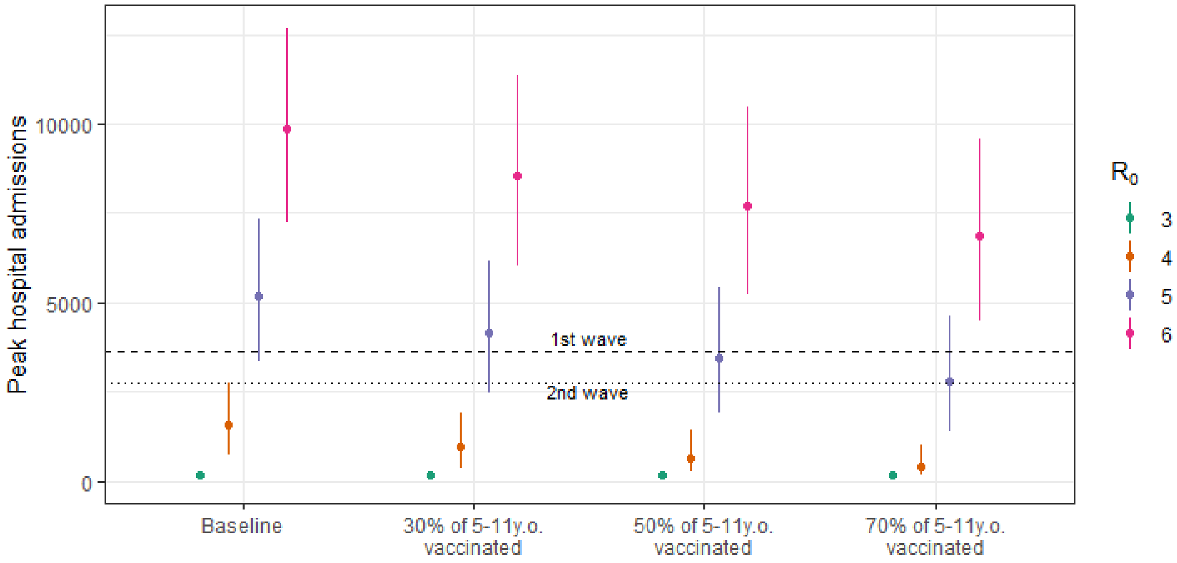

Figure S13: Projections in the absence of control measures, as a function of the basic reproduction number R_0 and different vaccine coverages in children aged 5 to 11y.o.. Peak in daily hospital admissions in the absence of control measures. This is done under the baseline assumption for the vaccine coverage (i.e. vaccine coverage of 70%-80%-90% among 12-17 y.o., 18-59 y.o. and over 60 y.o).

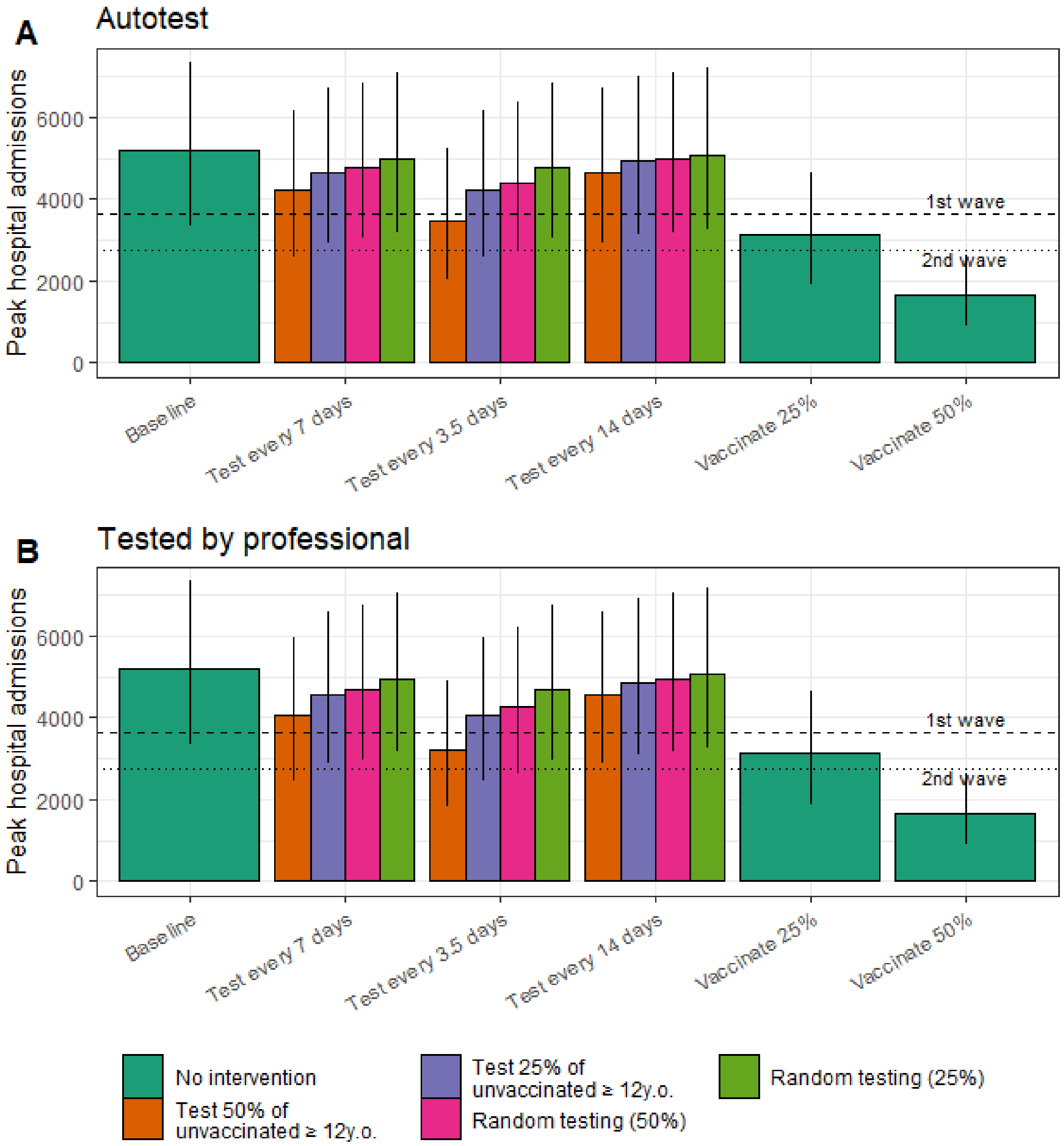

Figure S14: Peak in daily hospital admissions under different testing strategies. A. For self testing (sensitivity: 75%) **B.** For tests performed by a professional (sensitivity: 90%). The following interventions are explored: *Baseline* - no intervention; *Test every x days unvaccinated* - 50% or 25% of the unvaccinated individuals older than 12 y.o. are tested every x days; *Random* - the same number of individuals are tested but in the population of individuals older than 12 y.o. irrespective of vaccination status; *Vaccinate x%* - x% of the unvaccinated individuals older than 12 y.o. are vaccinated. Results are displayed for R_0=5.0. We assume 25% of the population has acquired protection through natural infection (range 20%-30% corresponding to the vertical bars).
